# Supplementary figures and images for: Phytolith profile of Acrachne racemosa (B. Heyne ex Roem. & Schult.) Ohwi (Cynodonteae, Chloridoideae, Poaceae)
Source: PLoS One. 2022 Feb 11;17(2):e0263721. doi: 10.1371/journal.pone.0263721 (PMC8836352; doi:10.1371/journal.pone.0263721)

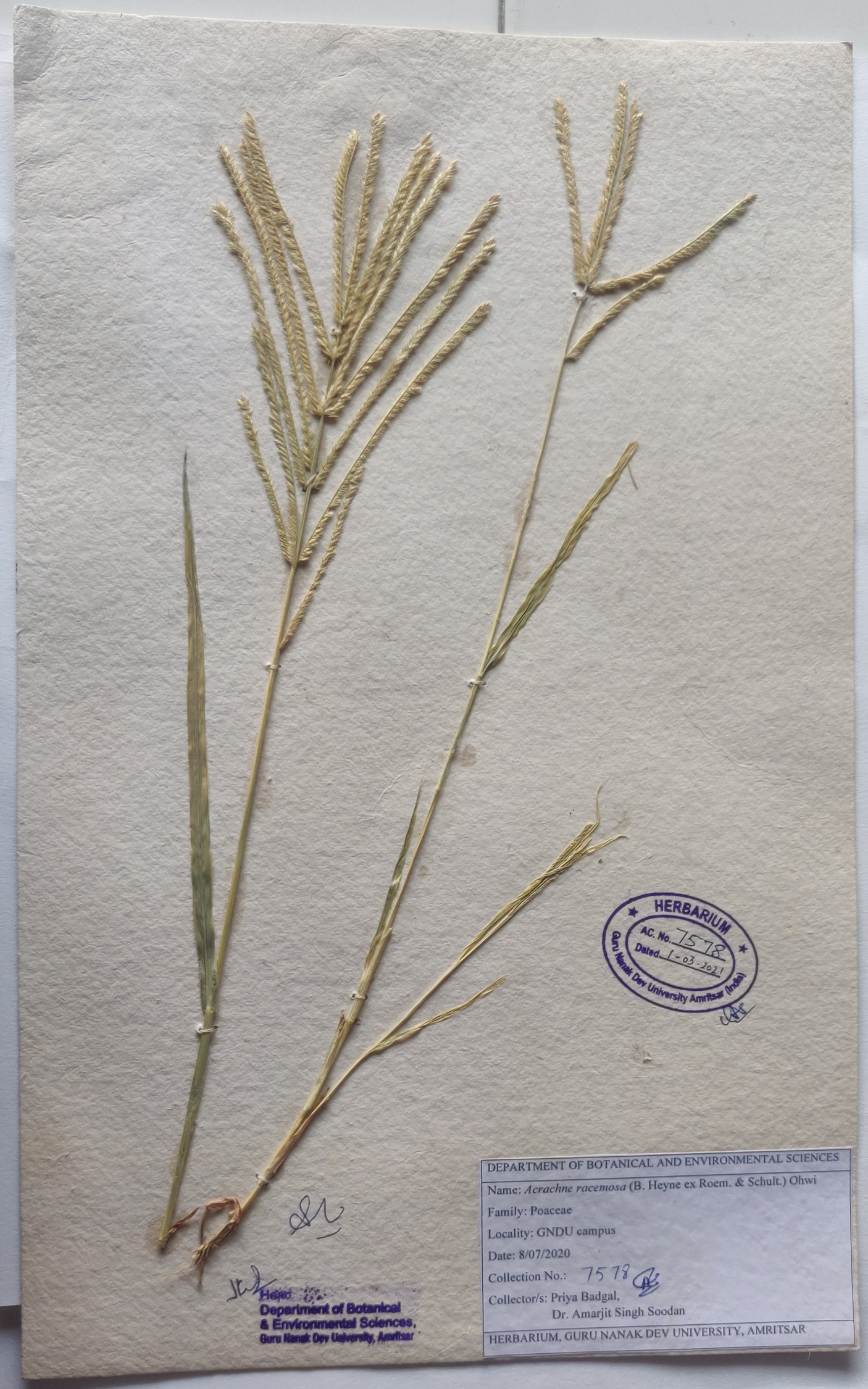

Supplement: S1 Fig — (TIF) [file pone.0263721.s001.tif]
