# Supplementary material for: Phytolith profile of Acrachne racemosa (B. Heyne ex Roem. & Schult.) Ohwi (Cynodonteae, Chloridoideae, Poaceae)
Source: PLoS One. 2022 Feb 11;17(2):e0263721. doi: 10.1371/journal.pone.0263721 (PMC8836352; doi:10.1371/journal.pone.0263721)
Supplement: S1 Table — (DOCX) [file pone.0263721.s002.docx]

**S1 Table:** FTIR peak showing different functional groups in phytoliths of *Acrachne racemosa* (Heyne ex Roem. & Schult.) Ohwi.

| **S.No.** | **PEAK POSITIONS**  **(Cm^-1^)** | **TYPE OF VIBRATION** | **STRUCTURAL UNIT** | **PLANT PARTS** | | | |
| --- | --- | --- | --- | --- | --- | --- | --- |
|  |  |  |  | **Root** | **Culm** | **Leaves** | **Synflorescence** |
| **1.** | 438.48-468.48 | **δO—Si---O** | --O--Si—O-- | + | + | + | + |
| **2.** | 665.94 | ν_s_Si—O--Si | ≡Si—O—Si≡ | ------ | ------ | + | ------- |
| **3.** | 786.94−801.46 | ν_s_Si—O | ≡Si—O—Si≡ | + | + | + | + |
| **4.** | 1058.93-1105.58 | **ν_as_Si—O--Si** | ≡Si—O—Si≡ | + | + | + | + |
| **5.** | 1633.69-1637-96 | **δH---O---H** | H---O---H | + | + | ------- | ------- |
| **6.** | 1875.88 |  | **Al_2_O_3_**.**SiO_2_** | + | ------- | ------- | ------- |
| **7.** | 2345.34-2362.47 | **ν_β_Si---C** | Si--R | + | -------- | + | + |
| **8.** | 3016.32 | νO---H | H—O—H…H_2_O | ------ | ------- | ------- | + |
| **9.** | 3419.76-3425.90 | O----H & Si----OH | H—O—H…H_2_O and ≡SiO---H….H_2_O | + | + | ------ | ------ |

ν= stretching vibration; νs=symmetric stretching vibration; ν_as_=antisymmetric stretching vibration; δ=deformation vibration; ν_β_=inplane stretching vibration
