# Supplementary material for: Phytolith profile of Acrachne racemosa (B. Heyne ex Roem. & Schult.) Ohwi (Cynodonteae, Chloridoideae, Poaceae)
Source: PLoS One. 2022 Feb 11;17(2):e0263721. doi: 10.1371/journal.pone.0263721 (PMC8836352; doi:10.1371/journal.pone.0263721)
Supplement: Attachment — Submitted filename: Response to Reviewer 2.pdf [file pone.0263721.s005.pdf]

PLOS ONE

Phytolith profile of Acrachne racemosa (Heyne ex Roem. & Schult.) Ohwi (Cynodonteae, Chloridoideae, Poaceae)

--Manuscript Draft--

**Manuscript Number:**	PONE-D-21-09972
**Article Type:**	Research Article
**Full Title:**	Phytolith profile of Acrachne racemosa (Heyne ex Roem. & Schult.) Ohwi (Cynodonteae, Chloridoideae, Poaceae)
**Short Title:**	Phytolith profile of Acrachne racemosa (Heyne ex Roem. & Schult.) Ohwi (Cynodonteae, Chloridoideae, Poaceae)
**Corresponding Author:**	PRIYA BADGAL, Ph.D<br>Guru Nanak Dev University Faculty of Life Sciences<br>Amritsar, Punjab INDIA
**Keywords:**	poaceae; phytoliths; morphotypes; morphometry; frequency; silica
**Abstract:**	Acrachne racemosa (Heyne. Ex roem. & Schult.) Ohwi of the subfamily Cynodonteae of the family Poaceae is an economically important plant. Phytoliths deposited in its inter and intracellular places by the accumulation of silica protects the plant from various types of stresses and provide strength and are helpful in the taxonomical studies. Phytolith morphotypes are useful for taxonomic identification of grass species and in the field of paleobotany, geology, and archaeology. In the present study, silica percentage was studied in different parts of the plant and maximum amount was found in leaf. Frequency and morphometric data of phytolith morphotypes from different parts of the plants were also collected and analysed. Various types of undulation were found in the fertile part. The strongest correlation was found between root and culm by Pearson's correlation coefficient which was supported by cluster analysis. Functional groups and crystalline polymorphic phases of silica were also analysed by FTIR and XRD. Saddle type of phytoliths had the highest frequency in leaf part and other forms of phytoliths in different parts of the plant were bilobate, blocky types, elongate types, trapezoids, triangular, cross, sinuate elongate, tabular types, nodular, cavate, scutiform, globular types. Globular types showed maximum roundness value and the elongate types shows maximum solidity value supported by Box-Whisker plot. From the present study, it was clearly understood that phytolith presence depend not only upon the morphological features but also on the anatomical and environmental factors. The paper aims at developing phytolith signatures of the species from various sites.
**Order of Authors:**	PRIYA BADGAL, M.Sc
	POONAM CHOWDHARY
	MUDASSIR AHMAD BHAT
	AMARJIT SINGH SOODAN
**Additional Information:**	
**Question**	**Response**
**Financial Disclosure**<br><br>Enter a financial disclosure statement that describes the sources of funding for the work included in this submission. Review the submission guidelines for detailed requirements. View published research articles from *PLOS ONE* for specific examples.<br><br>This statement is required for submission	The author(s) received no specific funding for this work.

and **will appear in the published article** if the submission is accepted. Please make sure it is accurate.<br><br>**Unfunded studies**<br>Enter: *The author(s) received no specific funding for this work.*<br><br>**Funded studies**<br>Enter a statement with the following details:<br>• Initials of the authors who received each award<br>• Grant numbers awarded to each author<br>• The full name of each funder<br>• URL of each funder website<br>• Did the sponsors or funders play any role in the study design, data collection and analysis, decision to publish, or preparation of the manuscript?<br>• **NO** - Include this sentence at the end of your statement: *The funders had no role in study design, data collection and analysis, decision to publish, or preparation of the manuscript.*<br>• **YES** - Specify the role(s) played.<br><br>* typeset	
**Competing Interests**<br><br>Use the instructions below to enter a competing interest statement for this submission. On behalf of all authors, disclose any competing interests that could be perceived to bias this work—acknowledging all financial support and any other relevant financial or non-financial competing interests.<br><br>This statement is required for submission and **will appear in the published article** if the submission is accepted. Please make sure it is accurate and that any funding sources listed in your Funding Information later in the submission form are also declared in your Financial Disclosure statement.<br><br>View published research articles from *PLOS ONE* for specific examples.	The authors have declared that no competing interests exist.

Powered by Editorial Manager® and ProduXion Manager® from Aries Systems Corporation

**NO authors have competing interests**<br><br>Enter: *The authors have declared that no competing interests exist.*<br><br>**Authors with competing interests**<br><br>Enter competing interest details beginning with this statement:<br><br>*I have read the journal's policy and the authors of this manuscript have the following competing interests: [insert competing interests here]*<br><br>* typeset	
**Ethics Statement**<br><br>Enter an ethics statement for this submission. This statement is required if the study involved:<br><br>• Human participants<br>• Human specimens or tissue<br>• Vertebrate animals or cephalopods<br>• Vertebrate embryos or tissues<br>• Field research<br><br>Write "N/A" if the submission does not require an ethics statement.<br><br>General guidance is provided below. Consult the submission guidelines for detailed instructions. **Make sure that all information entered here is included in the Methods section of the manuscript.**	Accession no. 915,<br>Department of Botanical and Environmental Sciences, Guru Nanak Dev University, Amritsar

Powered by Editorial Manager® and ProduXion Manager® from Aries Systems Corporation

**Format for specific study types**<br><br>**Human Subject Research (involving human participants and/or tissue)**<br>• Give the name of the institutional review board or ethics committee that approved the study<br>• Include the approval number and/or a statement indicating approval of this research<br>• Indicate the form of consent obtained (written/oral) or the reason that consent was not obtained (e.g. the data were analyzed anonymously)<br><br>**Animal Research (involving vertebrate animals, embryos or tissues)**<br>• Provide the name of the Institutional Animal Care and Use Committee (IACUC) or other relevant ethics board that reviewed the study protocol, and indicate whether they approved this research or granted a formal waiver of ethical approval<br>• Include an approval number if one was obtained<br>• If the study involved *non-human primates*, add *additional details* about animal welfare and steps taken to ameliorate suffering<br>• If anesthesia, euthanasia, or any kind of animal sacrifice is part of the study, include briefly which substances and/or methods were applied<br><br>**Field Research**<br><br>Include the following details if this study involves the collection of plant, animal, or other materials from a natural setting:<br>• Field permit number<br>• Name of the institution or relevant body that granted permission	
**Data Availability**<br><br>Authors are required to make all data underlying the findings described fully available, without restriction, and from the time of publication. PLOS allows rare exceptions to address legal and ethical concerns. See the PLOS Data Policy and FAQ for detailed information.	Yes - all data are fully available without restriction

A Data Availability Statement describing where the data can be found is required at submission. Your answers to this question constitute the Data Availability Statement and **will be published in the article**, if accepted.<br><br>**Important:** Stating 'data available on request from the author' is not sufficient. If your data are only available upon request, select 'No' for the first question and explain your exceptional situation in the text box.<br><br>Do the authors confirm that all data underlying the findings described in their manuscript are fully available without restriction?	
**Describe where the data may be found in full sentences. If you are copying our sample text, replace any instances of XXX with the appropriate details.**<br><br>• If the data are **held or will be held in a public repository**, include URLs, accession numbers or DOIs. If this information will only be available after acceptance, indicate this by ticking the box below. For example: *All XXX files are available from the XXX database (accession number(s) XXX, XXX.).*<br>• If the data are all contained **within the manuscript and/or Supporting Information files**, enter the following: *All relevant data are within the manuscript and its Supporting Information files.*<br>• If neither of these applies but you are able to provide **details of access elsewhere**, with or without limitations, please do so. For example:<br><br>*Data cannot be shared publicly because of [XXX]. Data are available from the XXX Institutional Data Access / Ethics Committee (contact via XXX) for researchers who meet the criteria for access to confidential data.*<br><br>*The data underlying the results presented in the study are available from (include the name of the third party*	All relevant data are within the manuscript.

and contact information or URL).<br>• This text is appropriate if the data are owned by a third party and authors do not have permission to share the data.<br><br>* typeset	
Additional data availability information:	

Manuscript Click here to access/download;Manuscript;MANUSCRIPT.docx

Phytolith profile of *Acrachne racemosa* (Heyne ex Roem. & Schult.) Ohwi (Cynodonteae, Chloridoideae, Poaceae)

[1]Priya Badgal*, [1]Poonam Chowdhary, [1]Mudassir Ahmad Bhat, and [1]Amarjit Singh Soodan
[1]Plant Systematics and Biodiversity Laboratory, Department of Botanical and Environmental Sciences, Guru Nanak Dev University, Amritsar (Punjab) India-143005.
E-mail: priyabadgal123@gmail.com*

Abstract

Acrachne racemosa (Heyne. Ex roem. & Schult.) Ohwi of the subfamily Cynodonteae of the family Poaceae is an economically important plant. Phytoliths deposited in its inter and intracellular places by the accumulation of silica protects the plant from various types of stresses and provide strength and are helpful in the taxonomical studies. Phytolith morphotypes are useful for taxonomic identification of grass species and in the field of paleobotany, geology, and archaeology. In the present study, silica percentage was studied in different parts of the plant and maximum amount was found in leaf. Frequency and morphometric data of phytolith morphotypes from different parts of the plants were also collected and analysed. Various types of undulation were found in the fertile part. The strongest correlation was found between root and culm by Pearson's correlation coefficient which was supported by cluster analysis. Functional groups and crystalline polymorphic phases of silica were also analysed by FTIR and XRD. Saddle type of phytoliths had the highest frequency in leaf part and other forms of phytoliths in different parts of the plant were bilobate, blocky types, elongate types, trapezoids, triangular, cross, sinuate elongate, tabular types, nodular, cavate, scutiform, globular types. Globular types showed maximum roundness value and the elongate types shows maximum solidity value supported by Box-Whisker plot. From the present study, it was clearly understood that phytolith presence depend not only upon the morphological features but also on the anatomical and environmental factors. The paper aims at developing phytolith signatures of the species from various sites.

keywords: poaceae, phytoliths, morphotypes, morphometry, frequency, silica

Introduction

Acrachne racemosa (Heyne ex Roem. & Schult.) Ohwi belongs to the genus *Acrachne* of subfamily Chloridoideae (family Poaceae) is an annual weed, sporadic monsoon grass commonly known as Chinkhe and is widely distributed in Pakistan, Punjab, N. W. F. P, Kashmir, Southeast Asia, tropical Africa, Ceylon and northern Australia [1]. *Acrachne racemosa* belong to tribe Cynodonteae that contain about 18 subtribes, 94 genera and 850

species [2]. The subfamily Chloridoideae includes about 5 tribes, 124 genera and 1602 species and are mainly found in arid temperate grassland or in arid tropical grasslands [3,4,5,2]. The grass family, Poaceae comprises of 768 genera and 11506 grass species [4,6,5,2]. It is a diverse and important family having great economic and ecological importance in every part of the earth [7,8]. The plants of this family have been used as stimulant and laxative, nerve tonic, diarrhea, dysentery, fever and cough and jaundice [9,10]. Grass epidermis, has three categories of elements namely bulliform elements, differentiated elements, fundamental elements [11]. About 20 years ago, *A. racemosa* was found in Kanchanaburi province, western Thailand and it has now been reported as a weed in upland crops like cassava, maize, sunflower and along the roads in several parts of the world. It grows in lawns, cultivated fields and waste places. The flowering and fruiting time of this plant is from July-September and is used in the treatment of piles and blood purification. This plant is traditionally used for diuretic purpose[12]. *A. racemosa* has the *in-vitro* antioxidant potential [1]. The grass species is an excellent fodder for cattle and the grain is never infected by insects.

Plants absorb silica in the form of orthosilicic acid along with water in the transpiration stream and by diffusion which induces root absorption [13]. Silicon is the second most abundant element in the earth's crust and is the eight most abundant element found in the universe [14,15] and represents more than 75% of the earth surface rocks [16] and is reported from sub-epidermal and epidermal layers of leaf and other parts including the protective covers in the glumes and lemmas and the epidermal layers of the caryopsis [17,18,19,20,21]. Adsorption of silicic acid is pH dependent [22]. Uptake of Silica (Si) achieved by three processes namely active, passive and rejective [23,13,24]. Through root, silica is translocated to the shoot along with water. Silicon contained in epidermal tissues, cell walls and phytoliths is from 15 to 79% [25]. It is an important element for various plant species [26,27,28,29] and plays a very important role in growth and development and protects the plant species from herbivores [30,30,31]. It offers resistence to lodging in plant species as it gives mechanical support due to attachment of Si with cell walls which provide rigidity to cell walls. Silica is deposited in amorphous hydrated silica bodies ($SiO_2.nH_2O$) called phytoliths or silica bodies or plant opal [32] which are rigid microscopic structures that develop in inter and intra cellular spaces of different organs namely culms, leaves, roots and synflorescence and may constitute upto 20% of the dry weight [33]. Phytoliths also contain small amount of Al, Ca, Fe, K, Mg, Mn, Na, and organic carbon (C) [34,35,36] along with

main components (SiO2 and H2O). Phytoliths are inorganic and resistant to decay and are durable fossil of terrestrial plants and after the decomposition of the plants, they are released into the soil [37,38,16,39]. They occur in all types of plants and their parts. Silica accumulates more in monocots and herbaceous species of families such as Poaceae, Cyperaceae, Commeliaceae and Zingiberaceae. More attention has been given to the family Poaceae [40,17]. The largest amount of Si is absorbed by sugarcane[41] (300-700 kg Si ha^{-1}), rice (150-300 kg Si ha^{-1}), wheat (50-150 kg Si ha^{-1}) [15]. In recent years, phytoliths analysis has progressed and extended our knowledge about microfossils (phytoliths) which are being used as palaeoenvironment and archaeological proxies [42]. Phytoliths are classified into two types (the cell wall type and the lumen type) depending on the place of deposition of silica [43,44,41,45] and they differs on the basis of plant genotypes and from the two, lumen type phytoliths dominate in the grass species[46,47,43,48,44,41]. Phytoliths shapes depends upon the size and shape of the host cell so it also varies [49,43,44,50]. Phytolith work is labour intensive and expensive like most archaeobotanical and paleoenvironmental work [51].

Phytoliths help the plant to resist abiotic (physical and chemical) and biotic stresses, diseases and pathogens [52,53] and help in the growth and development of plants [54] by providing stiffness, durability and mechanical support to the culm.. Physical stresses includes drought, radiation, high and low temperature. There is variation in distribution, diversity and frequency of opal phytoliths in the leaves, culm, root and synflorescence [33]. Types of phytoliths helps in the characterization of tribes and genera within Chloridoideae [39]. Information on the phytolith profiles of the foxtail grass genus and phytolith signature development provide evidence for the taxonomic demarcation [55]. The study of phytoliths helps taxonomists to identify grass species and thus useful for the taxonomic demarcation of the plant species. It helps in the detection of grasses and can distinguish C_3 grassland from C_4 grassland [56,57,20,58]. Phytoliths have been reported from the Late Devonian onwards [59]. Phytoliths were expected to give evidence of the regional climate [60]. On the basis of globular echinate phytolith a classification system was proposed which allowed the identification of tribes and subtribes of Amazonian palms [61].

Phytoliths been found have the potential to demarcate and identify some taxa and communities in the soil record under six community types in the Altay [62]. Study of bark phytoliths by [63] in African plants showed that there are number of taxonomically useful forms which were indicated good for future neotropical work. The silica microfossil record which was taken from the sedimentary sequence of the Pleistocene Holocene time period

from the Eastern Chaco Region showed that the most abundant elements found was phytoliths compared to other siliceous and non-siliceous microfossils [64]. Presence of silica skeleton and elongate dendritic phytolith has given evidence for the crop processing on the Taraschina site and the first analysis of phytolith which was conducted in the Danube Delta showed that Chalcolithic populations could grow cereal, 6000 years ago, in the heart of Delta [65]. [66] expressed that the spheroidal phytoliths in the roots of date palm look like the same or nearly so as echinates in the date palm aerial parts. It was suggested that the soil microaggregates which was the topsoil size fraction contribute for over 60 percent to the pool of phytoliths and control bioavailable Si and by trapping phytoliths protect them from rapid dissolution as the release of bioavailable Si is slower than that from the silt and clay fraction [67]. Phytoliths records a variety of taxa along with family, genus and might even possibly the species-level diagnostics of arboreal or woody plants along with ferns and underlying layer of vegetation in the wooden area of monocotyledon herbs in Neotrophical environment [68]. Six modern ecosystems were differentiated by soil phytolith assemblages in the ancient Maya tropical lowlands [69]. Phytolith analysis is a promising tool for the interpretation of the entire vegetation landscape and rapid changes in the northern temperate forest region corresponding well with the patterns evident in the pollen data which is stratigraphically equivalent. Fossil phytoliths gave additional information about changes in the C3/C4 plant ratios providing a new pespectives for the reconstruction of vegetation dynamics on the reliability of phytoliths in the northern temperate forest region [70]. These phytoliths extend our knowledge about microfossil and thus help the archeologists to recontruct the vegetations of grasses, palaeoenvironments and palaeoclimate as well as plant-people relationships [71,72,73,64,68,69,74].

The present study was designed to show the distribution of different phytolith types of *Acrachne racemosa*, its diversity, morphometric measurements and frequency present in different parts *viz*. root, culm, leaf and synflorescence. No work has been done on this species about the phytolith morphotypes including morphometric measurements and frequency as per our knowledge.

Material and Methods

Area of study

Acrachne racemosa (Heyne. Ex roem. & Schult.) Ohwi was collected from the campus of Guru Nanak Dev University (Amritsar) which is spread over an area of about 500 acres at 31.31ºN and 74.55 ºE. Species specimen has been submitted in the herbarium of Department of Botanical and environmental sciences, Guru Nanak Dev University, Amritsar with accession no. 915. Whole plant specimens were collected at the flowering stage along with the synflorescence. After taxonomic description, the specimens was divided into four fractions; culm, leaf and synflorescence (above ground) and roots (underground). And then for *in-situ* location of phytoliths, some of the dismembered parts were preserved in 70% ethanol at 4ºC . The remaining material was washed and then dried for dry ashing method.

Phytolith analysis

Phytoliths have become very important for field of taxonomic research during the last few years [20]. For study the morphological and distribution patterns of silica bodies, a number of techniques like Dry Ashing method, FTIR, XRD are being employed [32].

***In-situ* location (Epidermal peeling)**

The method of [75] with some modifications was used for *in-situ* location of phytolith in leaf samples from adaxial and the abaxial surfaces. Mature leaves were put under running tap water to remove dust and cut into segments which were boiled in distilled water for 5-10 minutes in test tubes (50ml). The test tubes containing leaf segments were removed from the water bath and left for cooling down. When they cooled down, distilled water was removed from the test tubes and leaf segments were put in fresh test tubes (50ml) containing ethanol (absolute) and heated gently (80◦C) in a water bath till green color was removed. Thereafter, in a solution of lactic acid and chloral hydrate (3:1 v/v), the segments were immersed and boiled again for 20–30 min in a water bath. Epidermal peeling became easy because lactic acid softened the leaf segment. After that, the leaf segments was taken out from the test tube, cleared, and was placed on the clean ceramic tiles with the abaxial surface upwards and then the epidermis was peeled off from the middle part of leaf blades. Therefore, in the same way peelings from adaxial surface of leaf segments were obtained. Thenafter, the peelings were stained in Gentian Violet and heated over in a watch glass over spirit lamb for deep staining. Finally, they was taken out and passed through a dehydration series of ethanol (30% through 50, 70, 90% and absolute ethanol) and then mounted in DPX for light microscopy and microphotography with a Micro Image Projection System (MIPS-USB 0262) mounted on an Olympus Binocular and connected to a computer for imaging.

Dry Ashing Method

For dry ashing of the plant material, the standard protocol of [49] was employed with some modifications. The plant specimens were separated into different parts: root, culm, leaves and synflorescence; they were washed under running tap water thoroughly to remove dust and were dried in an oven. They were cut into small pieces, weighed and transferred to porcelain crucibles which were put into the muffle furnace and incinerated at 550 ºC for 4-6 hours to ashes. After 4-6 hours, the crucibles were taken out of the furnace and then cooled and weighed. Then their ash contents were transferred to test tubes. The ash in the test tubes was submerged by sufficient quantity of hydrogen peroxide (30%) and the test tubes were kept at 80 ºC for 1 hour in a preheated water bath until the material settled down. Then the mixture was decanted off the hydrogen peroxide and the mixture rinsed twice in distilled water. Then HCl (10%) was added to the pellet and incubated for 1 hour at 80 ºC. Then the mixture was washed in distilled water and transferred to centrifuge tubes and centrifuged for 15 min at 7,500 rpm. The supernatant was decanted off and the pellet was washed in distilled water till the pellet was clear. Finally, the pellet was put in an oven and dried for 24 h at 60◦C to powder form and weighed. Then the powder was stored in an eppendorf and eppendorfs were put in a container.

The silica content (%)was calculated by the formula: ash content/dry mass x 100.

Photography, morphometric measurement and frequency

Five slides by weight were prepared for each sample by taking a small amount (ca. 0.1mg) of dried ash from the eppendorf and dipped in 10 ml of Gentian violet in a watch glass. Dry ash mixture in a drop of Gentian violet was put on a glass slide. Then the slides were heated gently and excess stain were drained off in the folds of a filter paper and slides were covered with a cover slip by putting DPX. Then the identification of morphotypes of phytoliths by their shape and size was done and frequency data of different morphotypes were taken. Mag Cam DC - 14 was used for photography of different morphotypes of phytoliths at a uniform magnification of 40X. The phytoliths isolated from underground (root) and aerial (culm, leaf, and synflorescence) parts by the dry ashing method showed

considerable diversity of phytolith morphotypes in terms of their shapes and were classified according to the International Code of Phytolith Nomenclature (ICPN 1.0 [76] ICPN 2.0[77].

Morphometry

To standardize the terminology used in the study of morphometry, ICPM (International Committee for Phytolith Morphometrics) was created and laid down four criteria for phytolith data i.e coherent, robust, reproducible and transferable [78]. Standardisation has fascilitated broadening of phytolith research [51]. Image J software (version 1.46r.) was used for morphometric measurements of morphotypes of phytolith. This software records dimensions and calculated values of size, surface area and shape including aspect ratio, roundness, solidity. PAST software (Paleontological Statistics software) was used to calculate Mean and standard error.

Extraction and Biochemical analysis

For the extraction of phytoliths, various procedures are used and to facilitate comparison we have to standardise the data [79-82]. While interpreting data, we should be aware of the depositional context and taphonomic issues [51]. FTIR (Fourier Transform Infrared) spectroscopy is a non-destructive and important analytical technique for researchers. FTIR analysis is accurate, rapid and a sensitive technique [83]. FTIR used satndard KBr method and provide IR spectra of silica powder produced by different parts of the *A. racemosa*. This results in the specific absorption or transmission of energy. XRD is also a rapid analytical technique which provide information on unit cell dimensions and is primarily used for phase identification of crystalline materials of *A. racemosa*. The graphs were made and data were analysed using origin pro 8.6 software and pcpdfwin software.

Results and Discussion:

Acrachne racemosa (Heyne. Ex roem. & Schult.) Ohwi is an annual grass having erect, simple or branched, tufted culms and glabrous compressed leaf sheath and other morphological features of this species are presented in Table 1. Identification of this grass has been done by using online sources; The Online World grass flore [84], GPWG [85], GPWG II [86].

Table 1. Morphological characters of *Acrachne racemosa* (Heyne ex Roem. & Schult.) Ohwi.

S.No	Characteristics	*Acrachne racemosa*
1.	Habit	Annual
2.	Culms	Erect or geniculately ascending
3.	Culm length	8-50 cm
4.	Leaf blade	Narrowly Lanceolate
5.	Length of leaf blades	7-20 cm
6.	Width of leaf blades	0.3-1.0 cm
7.	Leaf sheaths	Glabrous, compressed
8.	Leaf sheath margins	Glabrous
9	Inflorescence	Subdigitate or racemes
10.	Length of inflorescence	15 cm
11.	Spikelets	Oblong
12.	Spikelet length	6-10 mm
13.	Lower glume	Narrowly oblong
14.	Length of lower glume	1.2-3.0 mm
15.	Lower glume apex	Acute
16.	Upper glume	Lanceolate
17.	Length of upper glume	1.5-3.0 mm
18.	Upper glume apex	Acuminate
19.	Lemma	Ovate
20.	Lemma apex	Obtuse
21.	Lemma surface	Rugose
22.	Length of lemma	2-3 mm

Ash and Silica concentration

Acrachne racemosa deposits silica in the form of phytolith in different parts of the body. Dry Ashing method on different parts of the plant, revealed highest percentage of ash and silica accumulated in leaf (17.45% and 8.49%) followed by root (13.51% and 5.10%),

synflorescence (9.67% and 3.27%) and culm (8.36% and 1.31%) as shown in Fig. 1. Si content varies from 0.1 to 10% Si on a dry weight basis in plant shoots (Ma et al., 2001). Maximum amount of silica found in the leaf of grasses was also well reported [87,88,55]. Shoot parts show lower silica accumulation rate than the foliar parts of the pteridophytes [89].

Fig. 1 3D Column showing silica content in various parts of *Acrachne racemosa* (Heyne ex Roem. & Schult.) Ohwi.

In-situ location (Epidermal peeling)

After cytology, leaf epidermis plays the most important role in the taxonomy of plant species [90-96]. *In-situ* location of phytoliths in the leaf epidermis of *A. racemosa* revealed that phytoliths were presented in both abaxial and adaxial surfaces of leaf. [32] showed presence of silica bodies on both abaxial and adaxial surface of leaf epidermis of *K. macrantha*. Phytoliths give significant taxonomic information due to their consistent shape within species [97,20,98,99,55,100,101]. Bhat et al. [55] distinguish three species of the genus *setaria* on the basis of phytoliths pattern in abaxial and adaxial surface of the leaf. The short cells and the long cells are arranged in diagnostic patterns in the costal and intercostal regions of the leaf epidermis [40]. Abaxial leaf surface of *A. racemosa* showed 1-6 rows of saddle phytoliths arranged transversely in the costal region separated by short cells (Fig. 2A a-h, Table 2). Saddle-shaped phytoliths are the most taxonomically important phytolith among microcharacters as they were not found in any leaf surface of the genus of *Olyrinae* or *Parianinae* within *Olyreae* [102]. Prickle hairs were present on the costal region (Fig. 2A a). The costal rows of silica cells show the presence of oblong phytoliths (Fig. 2A c). It also shows the presence of elongate echinate phytolith along with saddles in the costal region (Fig. 2A d, Table 2). Cross shaped phytoliths are present in the costal region (Fig. 2A e). Bilobate type of phytoliths were present in the costal region (Fig. 2A f) separated by epidermal long cell. The intercostal region on abaxial surface lacked silica cells except the prickle hairs (Fig. 2A g). The phytoliths in costal and intercostal region of abaxial leaf surface included saddle, elongate echinate, bilobate, cross, prickle hair.

The adaxial surface had 1-10 rows of saddle phytoliths transversely arranged and separated by silica short cells in the costal region and very few of them in the intercostal region (Fig. 2B a-h). Prickle hair is found in the costal region on the adaxial surface (Fig. 2B

a). Saddle shaped phytoliths separated by epidermal long cells having an echinate outline were seen in costal region (Fig. 2B a-h). Elongate echinate with concave ends phytoliths were found in the intercostal region separated by stomata phytoliths (Fig. 2 B b) but were not found in the abaxial surface of the leaf layer and as per our knowledge present study is the first report of this type of phytolith in the adaxial surface of leaf epidermis of *A. racemosa*. The costal region shows saddle with notch shaped phytoliths and were separated by long epidermal cells with sinuate outline (Fig. 2B f). Cork cells were found in the costal region in the adaxial surface and were not found in the abaxial surface of leaf (Fig. 2B f). Elongate echinate phytoliths between saddles were found in the costal regions on the adaxial epidermal layer of *A. racemosa* (Fig. 2B h, Table 2). Short cells were found between long epidermal cells in the intercoastal region as shown in Fig. 2B. Adaxial leaf surface include saddles, saddles with notch, sinuate elongate and prickle hairs in very low frequency in inter-costal compared to costal region. Among the morphotypes, saddles had the highest frequency occurring in files upto 7 cells broad. In total, *A. racemosa* harboured seven foliar morphotypes. As we see prickle layer is present in the intercostal region in the abaxial surface while sinuate elongate are present in the intercostal region in the adaxial surface (Fig. 2A g, 2B b). It was seen that oblong phytoliths occur in costal region in the abaxial surface but not in the adaxial surface.(Fig. 2A c)

Fig. 2 ***Insitu*** **location of phytoliths in leaf epidermis of** ***Acrachne racemosa*** **(**Heyne ex Roem. & Schult.) Ohwi. A. Abaxial surface (a-h) B Adaxial surface(a-h) ; bar =40 μm

Table 2a: Morphometry of phytolith morphotypes of *Acrachne racemosa* (Heyne ex Roem. & Schult.) Ohwi *(Root)*

Phytolith morphotypes	Length(µm)	Width(µm)	Area(µm^2)	Perimeter(µm)	Aspect ratio(µm)	Round	Solidity
Acute Bulbous	-	-	-	-	-	-	-
Acicular	-	-	-	-	-	-	-
Arculate	-	-	-	-	-	-	-
Amoeboid	-	-	-	-	-	-	-
Bilobate	-	-	-	-	-	-	-
Blocky irregular	379.343±70.460	233.442±15.858	81314.39±19670.18	1140.244±165.658	1.520±0.140	0.674±0.059	0.937±0.004
Blocky polyhedral	402.748±72.924	343.178±75.985	126498.5±49729.32	1309.179±234.646	1.2042±0.086	0.845±0.050	0.941±0.006
Bulliform cells	288.182±43.156	171.654±27.003	41012.55±8661.031	817.675±99.196	1.688±0.201	0.632±0.084	0.929±0.016
Cross	-	-	-	-	-	-	-
Elongate	-	-	-	-	-	-	-
Elongate echinate	-	-	-	-	-	-	-
Globular granulate	190.610±34.975	156.807±30.891	26544.26±9373.74	586.945±101.186	1.224±0.053	0.822±0.034	0.9652±0.005
Globular polyhedral	369.901±49.715	324.382±40.243	104258.8±26316.2	1198.609±150.028	1.179±0.047	0.853±0.034	0.961±0.002
Globular echinate	-	-	-	-	-	-	-
Globular psilate	226.104±68.549	180.571±36.610	46335.78±17029.58	857.482±183.119	1.342±0.152	0.778±0.074	6.902±0.015
Nodular	-	-	-	-	-	-	-
Ovate	-	-	-	-	-	-	-
Parallelepipedal cell	-	-	-	-	-	-	-
Pentagon	-	-	-	-	-	-	-
Polylobate	-	-	-	-	-	-	-
Saddle	-	-	-	-	-	-	-
Sinuate elongate	-	-	-	-	-	-	-
Trapeziform	226.104±68.549	180.571±36.601	46335.78±17029.58	857.482±183.119	1.342±0.152	0.778±0.074	0.902±0.015
Triangular	227.623±5.833	171.0532±15.52819	36756.63±5520.581	797.595±81.609	1.314±0.127	0.788±0.071	0.093±0.026
Tabular simple	319.472±28.303	213.194±32.499	57094.5±12768.77	956.717±86.895	1.783±0.345	0.598±0.097	0.975±0.003
Tabular polyhedral	361.993±54.280	233.725±22.191	67548.51±11089.22	1083.532±93.538	1.381±0.179	0.751±0.105	0.920±0.018
Tabular irregular	282.372±11.527	170.795±23.987	40260.47±4905.078	849.67±68.127	1.621±0.274	0.660±0.130	0.93±0.019

#=mean±Standard Error: (-) = Absence of morphotype: *= very few morphotype

Table 2b: Morphometry of phytolith morphotypes of *Acrachne racemosa* (Heyne ex Roem. & Schult.) Ohwi *(Culm)*

Phytolith morphotypes	Length(µm)	Width(µm)	Area(µm^2)	Perimeter(µm)	Aspect ratio(µm)	Round	Solidity
Acute Bulbous	*	*	*	*	*	*	*
Acicular	-	-	-	-	-	-	-
Arculate	-	-	-	-	-	-	-
Amoeboid	589.708±55.912	303.736±23.261	145719.3±16786.71	2051.349±151.842	1.766±0.203	0.601±0.078	0.810±0.028
Bilobate	-	-	-	-	-	-	-
Blocky irregular	398.055±33.597	256.231±21.489	84656.54±10361.61	1335.117±169.238	1.6328±0.215	0.650±0.074	0.897±0.034
Blocky polyhedral	438.213±113.984	321.924±87.034	149452.8±66586.71	1391.069±401.813	1.366±0.093	0.744±0.044	0.905±0.022
Bulliform cells	163.513±16.872	96.628±11.871	14105.36±1816.47	526.007±32.829	1.415±0.122	0.728±0.064	0.869±0.008
Cross	-	-	-	-	-	-	-
Elongate	*	*	*	*	*	*	*
Elongate echinate	-	-	-	-	-	-	-
Globular granulate	155.218±53.456	192.501±60.293	33537.57±21492.96	635.614±208.892	1.221±0.090	0.832±0.060	0.951±0.006
Globular polyhedral	160.229±23.446	121.046±17.449	15545.01±3678.835	484.837±59.954	1.438±0.097	0.704±0.044	0.953±0.011
Globular echinate	240.331±16.582	204.532±13.714	43134.8±5028.326	792.066±41.644	1.140±0.004	0.877±0.003	0.968±0.003
Globular psilate	*	*	*	*	*	*	*
Nodular	615.903±90.236	251.554±18.375	121745.8±27855.08	1814.402±294.774	2.430±0.279	0.437±0.058	0.831±0.011
Ovate	161.679±61.404	92.008±18.967	15523.11±9336.89	461.313±161.987	1.673±0.208	0.617±0.081	0.963±0.005
Parallelepipedal cell	-	-	-	-	-	-	-
Pentagon	172.156±32.332	118.469±25.116	19122.98±6665.892	512.345±94.098	1.568±0.234	0.685±0.083	0.956±0.008
Polylobate	-	-	-	-	-	-	-
Saddle	-	-	-	-	-	-	-
Sinuate elongate	-	-	-	-	-	-	-
Trapeziform	124.561±34.594	70.781±11.188	10419.71±4775.453	395.057±92.285	1.592±0.215	0.670±0.081	0.935±0.013
Triangular	255.542±76.860	162.254±48.647	43906.77±22406.43	827.952±279.091	1.522±0.088	0.667±0.043	0.926±0.011
Tabular simple	*	*	*	*	*	*	*
Tabular polyhedral	-	-	-	-	-	-	-
Tabular irregular	-	-	-	-	-	-	-

#=mean±Standard Error: (-) = Absence of morphotype: *= very few morphotype

Table 2c: Morphometry of phytolith morphotypes of *Acrachne racemosa* (Heyne ex Roem. & Schult.) Ohwi (Leaves)

Phytolith morphotypes	Length(µm)	Width(µm)	Area(µm^2)	Perimeter(µm)	Aspect ratio(µm)	Round	Solidity
Acute Bulbous	378.957±25.391[#]	119.415±7.428	43856.43±2885.113	1160.483±72.710	2.863±0.390	0.378±0.053	0.824±0.028
Acicular	-	-	-	-	-	-	-
Arculate	*	*	*	*	*	*	*
Amoeboid	-	-	-	-	-	-	-
Bilobate	86.796±7.577	40.388±5.072	4365.505±499.403	281.597±17.948	1.800±0.094	0.562±0.030	0.903±0.007
Blocky irregular	*	*	*	*	*	*	*
Blocky polyhedral	*	-	-	-	-	-	-
Bulliform cells	214.037±23.049	153.705±20.898	28528.75±5352.749	657.435±70.862	1.362±0.125	0.759±0.068	0.938±0.007
Cross	68.724±8.620	36.653±2.519	3340.076±625.009	265.748±24.034	1.515±0.155	0.682±0.056	0.824±0.015
Elongate	476.720±60.180	49.558±3.616	23826.71±4035.366	1056.635±121.187	9.467±1.460	0.114±0.014	0.876±0.006
Elongate echinate	251.542±28.065	61.961±12.994	14604.16±4411.486	646.804±73.411	4.686±0.620	0.228±0.029	0.814±0.025
Globular granulate	134.595±10.933	121.762±12.008	13784.04±1749.663	444.86±28.536	1.098±0.038	0.912±0.032	0.963±0.002
Globular polyhedral	268.507±49.171	227.241±32.153	51808.01±14583.03	856.877±127.260	1.171±0.055	0.861±0.039	0.949±0.004
Globular echinate	*	*	*	*	*	*	*
Globular psilate	-	-	-	-	-	-	-
Nodular	-	-	-	-	-	-	-
Ovate	201.821±39.412	85.719±10.605	14873.88±4576.796	501.731±79.297	2.201±0.200	0.462±0.643	0.971±0.001
Parallelepipedal cell	234.032±18.687	162.536±13.025	34420.46±3931.019	743.445±43.405	1.537±0.184	0.687±0.077	0.963±0.006
Pentagon	-						
Polylobate	175.925±12.401	59.907±3.967	8923.322±380.834	482.932±10.400	3.088±0.564	0.343±0.053	0.863±0.009
Saddle	110.828±20.077	106.408±21.303	16920.86±5272.289	513.573±97.255	1.084±0.021	0.923±0.017	0.929±0.004
Sinuate elongate	652.793±134.616	86.639±14.851	54075.21±22211.24	1476.7±284.617	7.461±1.128	0.145±0.019	0.812±0.051
Trapeziform	153.4887±20.97947	136.633±22.150	18562.37±2518.617	593.912±41.263	1.7±0.230	0.608±0.072	0.913±0.016
Triangular	*	*	*	*	*	*	*
Tabular simple	291.878±78.0622	124.564±16.808	37172.43±10470.71	828.835±164.706	2.318±0.573	0.478±0.094	0.954±0.001
Tabular polyhedral	*	*	*	*	*	*	*
Tabular irregular	-	-	-	-	-	-	-

#=mean±Standard Error: (-) = Absence of morphotype: *= very few morphotype

Table 2d: Morphometry of phytolith morphotypes of *Acrachne racemosa* (Heyne ex Roem. & Schult.) Ohwi (Synflorescence)

Phytolith morphotypes	Length(μm)	Width(μm)	Area(μm²)	Perimeter(μm)	Aspect ratio(μm)	Round	Solidity
Acute Bulbous	475.017±89.370	138.412±12.883	40549.75±12277.54	1131.247±191.953	3.745±0.275	0.272±0.019	0.791±0.006
Acicular	251.436±42.469	42.618±2.676	7807.947±1713.322	559.121±94.103	5.2832±0.798	0.207±0.031	0.888±0.013
Arculate	685.896±107.358	68.154±16.334	42823.43±12292.53	1627.094±267.909	4.7674±0.447	0.216±0.018	0.457±0.072
Amoeboid	-	-	-	-	-	-	-
Bilobate	67.441±7.934	26.144±0.900	1925.924±352.913	198.546±21.364	2.586±0.179	0.393±0.025	0.861±0.015
Blocky irregular	424.939±49.466	305.5258±48.364	118380.5±27312.05	1330.752±147.855	1.3768±0.166	0.7604±0.070	0.948±0.010
Blocky polyhedral	389.202±110.292	269.9042±44.581	86575.67±37950.88	1151.341±251.176	1.5378±0.203	0.693±0.081	0.912±0.018
Bulliform cells	*	*	*	*	*	*	*
Cross	-	-	-	-	-	-	-
Elongate	721.449±205.067	84.544±13.016	63306.26±18802.41	1621.715±415.699	8.483±2.392	0.152±0.031	0.9±0.023
Elongate echinate	451.322±69.663	44.520±8.682	20501.01±4180.472	1147.957±155.0537	8.2302±0.849	0.1278±0.015	0.7148±0.013
Globular granulate	131.9734±20.972	107.2034±20.351	12644.06±3751.268	412.9834±67.857	1.2206±0.091	0.836±0.057	0.953±0.007
Globular polyhedral	-	-	-	-	-	-	-
Globular echinate	-	-	-	-	-	-	-
Globular psilate	-	-	-	-	-	-	-
Nodular	-	-	-	-	-	-	-
Ovate	-	-	-	-	-	-	-
Parallelepipedal cell	-	-	-	-	-	-	-
Pentagon	-	-	-	-	-	-	-
Polylobate	-	-	-	-	-	-	-
Saddle	57.175±2.500454	48.6364±2.588211	2826.613±139.4933	216.7358±5.612335	1.2124±0.1016679	0.8446±0.05992545	0.9302±0.01040865
Sinuate elongate	400.6545±52.64501	37.97875±1.063105	13274.18±1971.256	931.2642±125.0512	10.61075±1.447772	0.1±0.01487728	0.7735±0.02389386
Trapeziform	206.3398±67.49187	79.592±18.07434	21280.83±10294.77	618.607±164.6357	2.2916±0.4457386	0.5172±0.106629	0.8976±0.0193509
Triangular	*	*	*	*	*	*	*
Tabular simple	-	-	-	-	-	-	-
Tabular polyhedral	-	-	-	-	-	-	-
Tabular irregular	-	-	-	-	-	-	-

#=mean±Standard Error: (-) = Absence of morphotype: *= very few morphotype

Table 3. Type of phytolith morphotypes in Costal and Intercostal region of Abaxial and Adaxial leaf surface of *Acrachne racemosa* (Heyne ex Roem. & Schult.) Ohwi.

S.No.	Leaf surface	Region	Phytolith morphotypes
1.	Abaxial	Costal	1-6 rows of saddle phytolith
			Bilobate
			Oblong
			Cross
			Prickle hair
			Elongate echinate
		Intercostal	Prickle hair
			2 rows of stomatal cells
2.	Adaxial	Costal	1-10 rows of saddle phytoliths
			Saddle with notch
			Elongate echinate
			Prickle hair
		Intercostal	Elongate echinate with concave ends
			1-4 rows of stomatal cells

Phytolith morphotypes

Phytoliths have been reported mostly from aboveground parts like culm, leaf and synflorescences[11,103-108,33,109,39,110-114]. But in the present paper, we have increased the scope of phytolith analysis in Poaceae by study of phytoliths from the underground parts i.e (root) as well. [115] studied phytoliths from native plants as well as soil surface. Phytolith morphotypes play an important role in taxonomical demarcation as shown by three species of the foxtail genus which were classified on the basis of phytolith morphotypes [55]. About 42 phytolith morphotypes were identified in the present paper according to their diagnostic shapes and sizes. About twenty four (24) phytolith morphotypes were found in the underground part i.e root. About twenty three (23) and thirty nine (39) phytolith morphotypes were reported from the vegetative part i.e culm and leaves and around twenty (20) phytolith morphotypes along with undulation patterns were found in the fertile part i.e synflorescence. These morphotypes were divided into nine groups namely; blocky types, tracheids, short cells, long cells, epidermal elements, hairs, bulliform cells, globular types and tabular types. (Table 2 ; Fig. 3A-D). Blocky types of phytoliths originate from endodermis, tracheids from the vascular system and the remaining last seven morphotypes arise from the epidermis [11,107].

The long cells include sinuate elongate (Fig. 3C aae), elongate/saddle (Fig. 3C aaf), elongate echinate/saddle (Fig. 3C aag-aaf), smooth elongate with convex ends (Fig. 3C aaj), smooth elongate (Fig. 3C aak-aam; Fig. 3D z), arcuate (Fig. 3C aap), rough elongate (Fig. 3C aaq-aar), elongate echinate (Fig. 3C aat), elongate echinate with concave ends (Figure 3C aau), acute bulbous (Fig. 3A p; 3B u; 3C aav; Fig. 3D t-v), Long trapezoids (Fig. 3A l; 3B y,ab; 3D i), blocky cylindric (Fig. 3C aac-aad), blocky elongate (Fig. 3C aaa) and amoeboid. The short cells include saddle, cross, bilobate, polylobate, silica cork cell, reniform, saddle with notch, flat tower, crescent moon (Fig. 3C a-k; n-o; s-u; v; w; x; l-m; p-q; av), nodular (Fig. 3A w), horned tower (Figure 3B w-x), cavate (Fig. 3D f), blocky polyhedral (Fig. 3C ah, 3D q, 3A d), blocky irregular (Fig. 3D r-s, 3A u-v) globular psilate (Fig. 3D a, 3B b), globular polyhedral, globular granular, triangular, polyhedral, tabular irregular, tabular polyhedral (Fig. 3C am, o-p), tabular simple (Fig. 3A m; 3C ak-al, 3D g-h), cuboid, bulliform, clavate, pentagon, stellate (Fig. 3B n), prism shaped (Fig. 3A q-r). The bilobate, saddle type phytoliths were also reported by [33] in leaf sheath, leaf blade and culm of *Cynodon dactylon* (L.) Pers. by Laser Induced Breakdown Spectroscopy (LIBS). The saddle, bilobate, polylobate, cross and bulliform phytolith morphotypes of grasses were also recovered from lake Malawi sediments by [74]. Saddles are most common in xeromesophytes [62].

Some of the morphotypes were common to all parts of *Acrachne* spp (Table 2). The common morphotypes in all parts include acute bulbous (Fig. 3A p; 3B u; 3C aav; 3D t-v) Globular granulate (Fig. 3A b-c; 3B c-d; 3C ae-af; 3D b-c), trapezium (Fig. 3B y-z,aa; 3C ap, aw; 3D j) and triangular (Fig. 3A g-k; 3B h-i; 3C ai; 3D d-e). Unique phytoliths derived from various parts of *A. racemosa* included polylobate (Fig. 3C v), sinuate elongate (Figure 3C aae), flat tower (Fig. 3C p-q), oblong (Fig. 3C aa) and silica cork cell (Fig. 3C w) from leaf, horned tower (Fig. 3B w-x) from the culm, prism shaped (Fig. 3A q-r) and nodular (Fig. 3A w) from root, saddle (Fig. 3C a-k) phytoliths from leaf and synflorescence (within undulations as shown in Fig. 3E), saddle with notch (Fig. 3C l-m) from leaves, bilobate (Figure 3C r-u), elongate echinate (Fig. 3C aar-aau) and acute bulbous (Figure 3C aav; 3D t-v) from leaf and synflorescence, reniform (Fig. 3C x), arcuate (Fig. 3C aap), tracheid (Fig.3C ax) and crescent moon (Fig. 3C av) from leaf, ovate (Fig. 3C y-z) from leaf and synflorescence, cuneiform bulliform (Fig. 3A y; 3B p-r; 3C aq-ar) from root, culm and leaf, cavate (Fig. 3A s; 3B s; 3D f) from root, culm and synflorescence, acute (Fig. 3D w-x) and acicular (Fig. 3D y) from synflorescence, cross shaped (Fig. 3C n-o), smooth elongate (Fig.

3C aaj-aan; Fig. 3D z) phytoliths from leaf and synflorescence, Scutiform (Figure 3C ay), blocky elongate (Fig. 3C aaa), blocky cylindrical (Fig. 3C aac-aad), elongate/saddle (Fig.3C aaf), elongate echinate/saddle (Fig. 3C aag-aai), rough elongated (Fig. 3C aaq-aar) from leaf, elongate irregular (Fig. 3B af; 3C az, aao) from culm and leaf, tabular polyhedral (Fig. 3A v; 3C am; 3D o-p;), blocky polyhedral (Fig. 3A t; 3C ah; 3D l) and tabular simple (Fig. 3A m; 3C ak-al; 3D g-h) from root, leaf and synflorescence, blocky irregular (Fig. 3A u; 3D r-s) from root and synflorescence, polyhedral (Fig. 3B m; 3C aj; 3D m-n;) from culm, leaf and synflorescence, ellepsoidal (Fig. 3C ab-ad) from leaf, globular echinate (Fig. 3B e-f) from culm.

Fig. 3A. Phytolith morphotypes of *Acrachne racemosa* (Heyne ex Roem. & Schult.) Ohwi (Root): Globular psilate (a); Globular granulate (b-c); Blocky polyhedral (d); Globular polyhedral (e-f); Triangular (g-k); Long trapezoids (l); Tabular simple (m); Trapezoid (n-o); Acute bulbous (p); Prism (q-r); Blocky polyhedral (s-t); Blocky irregular (u-v); Nodular (w); Cuneiform bulliform (x-y).

Fig. 3B. Phytolith morphotypes of *Acrachne racemosa* (Heyne ex Roem. & Schult.) Ohwi (Culm) : Orbicular (a); Globular psilate (b); Globular granular (c-d); Globular echinate (e-f); Globular polyhedral (g); Triangular (h-i); Polygonal (j); Pentagon (k-l); Polyhedral (m); Stellate (n); Flabellate bulliform (o-p), Cuneiform bulliform (q-r); Cavate (s); Cuneiform (t); Acute bulbous (u); Trapezoid (v); Horned tower (w-x), Long trapezoid (y, ab); Tabular simple (z); Trapezoid (aa); Cuboid (ac-ad); Smooth elongate (ae); Elongate irregular (af). bar=20 µm

Fig. 3C. Phytolith morphotypes of *Acrachne racemosa* (Heyne ex Roem. & Schult.) Ohwi (Leaves): Saddle (a-k); Saddle with notch (l-m); Cross (n-o); Flat tower (p-q); Bilobate (r-u); Polylobate (v); Silica cork cells (w); Reniform (x); Ovate (y); Oblong (z); Ellipsoidal (aa-ad); Globular granulate (ae-af); Globular polyhedral (ag); Blocky polyhedral (ah); Triangular (ai); Polyhedral (aj); Tabular simple (ak-al); Tabular polyhedral (am); Cuboid (an-ao); Trapezoids (ap); Cuneiform bulliform (aq-ar); Carinate (as); Scutiform (at-au); Crescent moon (av); Rectangular (aw); Elongate echinate (ax); Scutiform (ay); Elongate irregular (az); Blocky elongate (aaa); Blocky cuboid (aab); Blocky cylindrical (aac-aad); Sinuate elongate (aae); Elongate/Saddle (aaf); Elongate echinate/Saddle (aag-aai); Smooth elongate with convex ends (aaj); Smooth elongate (aak-aan); Elongate irregular (aao); Arcuate (aap); Rough elongate (aaq-aar); Elongate echinate (aar-aat); Elongate echinate with concave ends (aau); Acute Bulbous (aav). bar= 10 µm (a-ab, aaj-aav); 40 µm (ac-aai)

Fig. 3D. Phytolith morphotypes of *Acrachne racemosa* (Heyne ex Roem. & Schult.) Ohwi (Synflorescence): Globular psilate (a); Globular granulate (b-c); Triangular (d-e); Cavate (f); Tabular simple (g-h); Long trapezoids (i); Trapezoid (j); Polygonal (k); Blocky polyhedral (l); Polyhedral (m-n); Tabular polyhedral (o-p); Blocky polyhedral (q); Blocky irregular (r-

s);Acute bulbous (t-v) Acute (w-x) Acicular (y); Smooth elongate (z); Elongate echinate (aa): bar=20 µm

Undulation patterns

Due to the differences in the undulation amplitude, there is differences in the morphology of the undulated patterns among Ω type, η type, and β type and three important parameters were used to characterize the morphological variations of the structures of the β-type undulated patterns [116]. β-shaped undulation patterns from the inforescences was also found by [117]. [118] classified undulation according to the type of undulation pattern, main body and connection. Undulated patterns and ornamentations were seen in the fertile part (synflorescence) of *Acrachne racemosa* (Fig. 3E). It showed elongate granulate and bilobate phytoliths were embedded in it (Fig. 3E a), It also shows elongate collumellate extensions on the epidermal long cells with embedded bilobates (Fig. 3E b), with embedded cork cells (Fig 3Ec). With embedded prickle hair and bilobates, elongate granulate type of undulation were also found (Fig. 3E d-e). It also shows the presence of elongate granulate type of margin on the epidermal long cell along with saddles (Fig. 3E f), cross (Fig. 3Eh). Elongate clavate along with bilobates were also seen on the epidermal long cell of synflorescence (Fig. 3Eg). Elongate castellate type of ornamentation were also seen along with saddle (Fig. 3E i) and stomata (Fig. 3E j).

Fig. 3E. Undulated patterns in *Acrachne racemosa* (Heyne ex Roem. & Schult.) Ohwi (Synflorescence); Elongate granulate/bilobates (a); Elongate columellate/biolobate (b); Elongate Columellate/cork cells (c); Elongate granulate/bilobates/ prickle hair (d-e); Elongate granulate/saddle (f); Elongate clavate/bilobates (g); Elongate granulate/cross (h); Elongate castellate/stomata/saddle (i); Elongate castellate /stomata (j). bar=40 µm

Frequency distribution and morphometric measurements

The data on frequency distribution and morphometric measurements of phytolith morphotypes increase the knowledge and helps in the identification of the species [119,110,98,99,55,114,115]. The frequency of morphotypes shows variations in different parts of the plant species. In underground part (root), blocky polyhedral has the highest

frequency (20.22%) followed by blocky irregular (15.39%), globular granulate (8.52%), globular polyhedral (5.85%) whereas oblong, acute bulbous, cuboid and prism present in low frequencies. Nodular phytolith present only in roots with 0.76 % frequency. Mostly blocky types of phytoliths are found in roots (Fig 3A) and represent loss of scutiform phytoliths. Blocky irregular (11.7%) constitute the highest frequency in the culm part followed by blocky polyhedral (7.35%), triangular (5.68%), globular granular (5.35%) whereas tabular simple, scutiform, stellate possess low frequency. Stellate type of phytolith found only in the culm with the absence of acicular, clavate, oblong, flat tower. In vegetative part (leaf) saddle phytoliths were the most common category accounting for more than 40% of the total phytoliths and constitutes the highest frequency in leaf part followed by smooth elongate (7.66%), elongate echinate (5.75%) and bilobate (3.40%) with a very low frequency of reniform (0.04%) and carinate (0.08%) with the absence of tabular irregular, stellate and nodular type of phytoliths. Saddle-shaped phytoliths are known to be the diagnostic for members of subfamily Chloridoideae. In fertile part (synflorescence), undulations patterns complexed with different morphotypes has the highest frequency with a low frequency of ovate, pentagon and acute. Saddle phytoliths are also present in synflorescence with very less frequency (2.14%) but are absent in the root and culm part of the species. The frequency of elongate echinate is the highest in synflorescence (8.56%) followed by leaf (5.75%), culm (1.00%) but are not found in roots. Their is absence of cuneiform bulliform, rectangular, cuboid but are found in other parts of *Acrachne racemosa.* Comparison of shared phytoliths in different parts i.e root, culm, leaves and synflorescence also show significant differences. The frequency of smooth elongate is very less in root (0.63%) as compared to leaf (7.66%), culm (2.67%) and synflorescence (2.33%). The frequency of trapeziform were lesser in synflorescence as compared to the culm, root and leaf. Globular granular phytolith had high frequency in root (8.52%) as compared to culm (5.35%), synflorescence (3.89%) and leaf (2.34%). Blocky irregular contributes the highest frequency in roots then in culm. Blocky polyhedral also contributes highest frequency in roots (20.22%) then culm (7.35%). Triangular phytoliths has the maximum frequency in culm then root, leaf and synflorescence (Table 4).

Table 4. Frequency distribution of phytolith morphotypes from different parts of the *Acrachne racemosa* (Heyne ex Roem. & Schult.) Ohwi.

S.No.	Phytolith morphotype	Root (%)	Culm (%)	Leaf (%)	Synflorescence(%)
1.	Acicular	0.25	---	0.25	2.72

2.	Acute bulbous	0.38	0.66	1.02	7.58
3.	Arcuate	---	---	0.04	---
4.	Bilobate	---	----	3.40	---
5.	Blocky irregular	15.39	11.70	0.55	1.16
6.	Blocky polyhedral	20.22	7.35	0.21	0.38
7.	Clavate	0.38	---	0.12	---
8.	Crescent moon	---	---	0.04	---
9.	Cross	---	---	0.04	---
10.	Cuboid	1.27	2.00	0.38	----
11.	Cuneiform	0.50	2.00	0.12	0.19
12.	Cuneiform bulliform	3.56	3.34	0.08	---
13.	Ellepsoidal	8	---	3	---
14.	Elongate echinate	---	1.00	5.75	8.56
15.	Elongate irregular	---	1.00	0.38	1.55
16.	Flat tower	---	---	0.85	---
17.	Globular echinate	1.27	0.66	0.21	0.58
18.	Globular granulate	8.52	5.35	2.34	3.89
19.	Globular polyhedral	5.85	3.01	0.51	0.19
20.	Globular psilate	2.16	3.01	0.04	0.58
21.	Horned tower	---	1.00	0.59	---
22.	Oblong	---	---	0.25	---
23.	Oval	---	---	0.76	---
24.	Ovate	3.68	0.33	0.89	0.19
25.	Parallelepipedal cell	0.76	1.00	0.59	0.38
26.	Polyhedral	3.30	0.33	0.55	0.77
27.	Polylobate	---	---	0.72	---
28.	Prism	0.25	---	---	---
29.	Rectangular	2.41	3.34	1.19	---
30.	Reniform	---	---	0.04	---
31.	Saddle	---	---	48.95	4.28
32.	Saddle with notch	---	---	0.29	---
33.	Scutiform	---	0.33	0.17	0.58
34.	Sinuate elongate	---	---	1.91	2.91
35.	Smooth elongate	0.63	2.67	7.66	2.33
36.	Stellate	---	0.66	---	---
37.	Tabular simple	3.05	0.66	0.17	---
38.	Tabular irregular	0.76	11.03	---	---
39.	Tabular polyhedral	4.45	2.00	0.08	0.38
40	Trapezium	2.79	3.67	2.0	0.58
41.	Triangular	3.68	5.68	0.93	0.58
42.	Nodular	0.63	---	---	---

Morphometric data on size dimensions (length, width, area and perimeters) and shape descriptors (aspect ratio, solidity and roundness) of morphotypes used for the taxonomic resolution of plant species [120,108,55]. Morphometry of phytolith morphotypes that were

present in high frequency were calculated (Table 2; Fig. 4). Data of size dimensions and shape descriptors have been collected in our present study. Data of shape descriptors gives quantitative values as shown in Figure 4 which helps in the variability of data. Solidity is a measure of the ratio between the total surface area to the convex area of a type and roundness value is unity for perfect circles and with an increase it decreases along any of the dimensions. When a particular morphotype is longer than wide, it shows that aspect ratio>unity and ranges from >1-3 for short cell types and >5 to <11 for long cell types. The roundness value of elongate echinate, smooth elongate, sinuate elongate does not exceed 0.30 (Fig. 4 c, d) and thus showed minimum roundness value. Roundness value of elongate types hardly exceeded 0.60 [98]. The globular polyhedral (Fig. 4a, c) triangular, trapezoid (Fig. 4a), ovate (Fig. 4c), saddle (Fig. 4c,d) and globular granular (Fig. 4d) types of phytoliths showed maximum roundness values of >0.9. The globular psilate and tabular simple showed the maximum solidity value as seen in Box-Whisker plots (Fig. 4e) whereas the minimum solidity value were shown by amoeboid and arculate (Fig. 4h and f) type of phytoliths.

Fig. 4. **Box-Whisker plots for median percentile and range of shape descriptors (roundness and solidity) of phytoliths in *Acrachne racemosa*** (Heyne ex Roem. & Schult.) Ohw

Correlation analysis among morphotypes of phytolith from different parts of *A. racemosa* based on the pearson's coefficient showed positive and negative correlation (Table 5). The strongest association was found between root and culm and the lowest between leaf and culm. The association between root and culm is more significant than others. Root and culm shows the positive association while root and synflorescence have the negative association with each other. Leaf is positively associated with synflorescence. Leaf is more associated with synflorescence than root and culm and root is more associated with culm than leaf and synflorescence. This shows that silica decomposition depend upon the internal structures, physiology and on the environmental factors (Table 5). On the basis of jaccard's similarity index, cluster diagram of phytolith morphotypes of species showed that 67% of similarity was found between root and culm and were grouped together whereas around 58% of similarity was found between root, culm and synflorescence. However, all parts of the *Acrachne racemosa* showed 55% of similarities . Maximum similarity was found between root and culm (Fig. 5).

Fig. 5. Clustering of *Acrachne racemosa* (Heyne ex Roem. & Schult.) Ohwi based on presence/absence data of phytolith morphotypes. R=root, C=culm, L=leaf and S=synflorescence

Table 5: Coefficient correlation among the phytolith morphotypes from different parts of *Acrachne racemosa* (Heyne. Ex roem. & Schult.) Ohwi based on the Pearson's coefficient.

**Species**		***Acrachne racemosa***			
		**Root**	**Culm**	**Leaf**	**Synflorescence**
Acrachne racemosa	**Root**	1	0.8092	-0.0756	-0.1103
	**Culm**	0.8092	1	-0.0660	-0.1238
	**Leaf**	-0.0756	-0.0660	1	0.1150
	**Synflorescence**	-0.1103	-0.1238	0.1150	1

Biochemical architecture

FTIR

IR Spectra of silica powder were obtained using the standard KBr method on a FTIR at room temperature. This technique is used for chemical characterization of the biological samples. Through this technique the purity of phytoliths has been examined [121][122][123]. Phytolith functional groups from different parts of plant species were known by FTIR analysis. Over wavelength range of 400-4000 cm^{-1}, the spectra of functional group were marked. FTIR spectra of silica from root, stem, leaves and synflorescence of *Acrachne racemosa* showed several peaks which were assigned to various structural units of silica. The wide peak is seen at 1088.94 cm^{-1} in root and the longest peak is seen at 1058.93 cm^{-1} in leaf. The peaks between 438.48-468.48 cm^{-1} (Fig. 6) present in root, culm, leaf and synflorescence parts of the *Acrachne racemosa* has been ascribed to deformation vibration of O-Si-O group [124] bonds. The peaks at 665.94 cm^{-1} has been assigned to a symmetrical vibration of Si-O-Si [125] bonds present only in culm. The peaks between 786.94-795.65 cm^{-1} has been assigned to symmetrical vibration of Si-O [126] bonds are found only in root and culm but not in leaf and synflorescence. The peaks between 800.82-801.46 cm^{-1} is the characteristics of Si-O-Si in Siloxane group could be ascribed to symmetric streching mode [127][128] are present only

in leaf and fertile part i.e synflorescence. The peak between 1058.93-1105.58 cm^{-1} correspond to asymetric vibration of Si-O-Si [129,130,128] bonds. The peaks between 1757.60-1875.88 cm^{-1} has been assigned to deformation vibration of alkyl (R group) are found only in underground part (root) and fertile part (synflorescence). The peaks between 1633.69-1637-96 cm^{-1} present only in root and culm could be ascribed to deformation of H-O-H [131]bonds. The peaks between 2345.34-2362.47 and 3419.76-3425.90 correspond to inplane stretching vibration of Si-C and O-H/Si-OH bonds [126] bonds.

Fig 6. FTIR spectra of phytoliths from different parts of *Acrachne racemosa* (Heyne ex Roem. & Schult.) Ohwi

XRD analysis

Due to high temperature (550 C for 4-6 h) during Dry Ashing Method, amorphous silica gets transformed into the crytalline form. Several polymorphic phases were seen depending upon the temperature and pressure [132]. XRD diffraction of phytoliths isolated from different parts of *Acrachne racemosa* showed peaks characteristics of different crystalline polymorphic phases of silica that include Quartz, cristobalite alpha, tridymite (from the steinbach r), Quartz low, coesite, stishovite, non-ambient pressure (Fig. 7). These phases have identical chemical composition (SiO_2) but different lattice systems including anorthic, monoclinic, orthorhombic, flexagonal, hexagonal and tetragonal. Present study shows that tetragonal body centered phase occurs only in culm not in root, leaf and synflorescence which gives credence to the existence of polymorphic silica in plants [20,107,133,134]. XRD analysis of silica extracted from the grass species reported the presence of coesite, cristobalite and tridymite [135,136]. Monoclinic primitive and hexagonal at non-ambient pressure phase were found only in synflorescence while orthorhombic body-centered and orthorhombic tridymite end-centered occurred in the root. Tridymite is derived from oxidation of fayalite [137]. Peaks corresponding to tetragonal cristobalite alpha and anorthic primitive were found in all parts of the species while peak corresponding to monoclinic end-centered was found in culm and root. Hexagonal quartz primitive were diagnostic of root, culm and synflorescence. Hexagonal quartz low primitive, hexagonal primitive, monoclinic coesite end-centered and tetragonal stishovite primitive were present in root and synflorescence.

Fig 7. XRD diffraction spectra of phytoliths isolated from different parts of *Acrachne racemosa* (Heyne ex Roem. & Schult.) Ohwi

Conclusion:

Chinkhe shows a high frequency of saddle phytoliths that are transversely arranged in costal area in the adaxial and abaxial region of the epidermal layer. Prickle layer is present in the intercostal region in the abaxial surface while sinuate elongate are present in the intercostal region in the adaxial surface. Phytoliths isolated from aboveground and underground parts by Dry Ashing Method show variety of phytolith morphotypes in terms of shape and size. Phytoliths present in roots are mostly blocky types. Various types of undulation patterns and ornamentations were seen in phytoliths from fertile part i.e synflorescence. Vegetative part (leaf) possess highest amount of silica and ash followed by underground part (root) then fertile part (synflorescence). Least amount of silica possessed by culm. Variation in silica amount, morphotypes, morphometric measurement and frequency depending on various factors involving anatomical, physiological and environmental could be used for the identification and taxonomic demarcation of the species. Polylobate and cross shaped type of phytoliths are characteristically found in leaf and Box-Whisker plots showed that Globular psilate and tabular simple possessed maximum solidity value. Blocky polyhedral possess highest frequency in root, blocky irregular in culm, saddle in leaf and undulations patterns complexed with different morphotypes has the highest frequency in synflorescence. Maximum similarity was found between root and culm. Phytoliths provide resistance and help the plant species in growth and development by protecting it from various stresses and play a very important role in research by acting as a tool for phytolith analysis in environmental and systematic biologists, geologists paleobotanists and archeologists and help in the reconstruction and interpretation of the vegetation of the grass species. FTIR analysis showed various modes of vibrations. XRD diffraction of phytoliths showed peaks characteristics of different crystalline polymorphic phases of silica. The phytolith signatures of species developed in the present work shall provide additional parameters for identification and diagnosis of the *Acrachne racemosa*.

Acknowledgments

The authors are thankful to head of the department of Chemistry, Guru Nanak Dev University, Amritsar for providing XRD and FTIR instrumentation facility. We also thank Mr. Shakoor for his help in XRD analysis.

Author Contribution

PB carried out experimental work and data analysis and wrote the initial draft of this manuscript. PC helped in FTIR analysis. MAB identified the plant specimen. ASS conceived the work idea and also supervised the work and drafted the final manuscript. All authors have equally contributed in the writing of the manuscript.

Competing Interests

The authors have no competing interest.

References

1. Athar Abbasi M, Nazir K, Shahzadi T, Riaz T, Zahra Siddiqui S, Ajaib M. Acrachne racemosa: A Potent Source for Natural Antioxidants. 2011.
2. Soreng RJ, Peterson PM, Romaschenko K, Davidse G, Teisher JK, Clark LG, et al. A worldwide phylogenetic classification of the Poaceae (Gramineae) II: An update and a comparison of two 2015 classifications. Journal of Systematics and Evolution. Wiley-Liss Inc.; 2017. pp. 259–290. doi:10.1111/jse.12262
3. Clayton W, the SR-G graminum. G of, 1986 undefined. Genera graminum. Grasses of the world. cabdirect.org. [cited 20 Mar 2021]. Available: https://www.cabdirect.org/cabdirect/abstract/19871657525
4. Watson L, Dallwitz MJ. The grass genera of the world. The grass genera of the world. 1992.
5. Soreng RJ, Peterson PM, Romaschenko K, Davidse G, Zuloaga FO, Judziewicz EJ, et al. A worldwide phylogenetic classification of the Poaceae (Gramineae). Wiley Online Libr. 2015;53: 117–137. doi:10.1111/jse.12150
6. GPWG (Grass Phylogeny Working Group II). (2011).... - Google Scholar. [cited 20 Mar 2021]. Available: https://scholar.google.co.in/scholar?hl=en&as_sdt=0%2C5&q=GPWG+%28Grass+Phylogeny+Working+Group+II%29.+%282011%29.+New+grass+phylogeny+resolves+deep+%09evolutionary+relationships+and+discovers+C4+origins.+New+Phytol.+193%2C+304–312.+doi%3A+%0910.1111%2Fj.1469-8137.2011.03972.x&btnG=
7. Khan M, Ali S, Yaseen T, … SU-RJ of, 2019 undefined. Eco-Taxonomic Study of Family Poaceae (Gramineae). jbas.juw.edu.pk. [cited 20 Mar 2021]. Available: http://jbas.juw.edu.pk/index.php/JBAS/article/view/191

8. Samreen U, Ibrar M, in LB-CO, 2015 undefined. Ethnobotanical usages of Poaceae family in district Bannu. search.proquest.com. [cited 20 Mar 2021]. Available: https://search.proquest.com/openview/339ed72fde7fc292f79359c899459527/1?pq-origsite=gscholar&cbl=2032489

9. Jan G, Ajab Khan M, Gul F, Ahmad M, Jan M, Zafar M. ETHNOBOTANICAL STUDY OF COMMON WEEDS OF DIR KOHISTAN VALLEY, KHYBER PAKHTOONKHWA, PAKISTAN. Pak J Weed Sci Res. 2010. Available: http://www.wssp.org.pk/weed/ojs/index.php/pjwsr/article/view/301/301

10. Kamal M, Wazir S, Hassan M, Subhan M, … SK-PJP, 2009 undefined. Ethnobotanically important plants of district Bannu, Pakistan. academia.edu. [cited 21 Mar 2021]. Available: https://www.academia.edu/download/46572352/ETHNOBOTANICALLY_IMPORTANT_PLANTS_OF_DIS20160617-22781-x97jjb.pdf

11. Twiss PC, Suess E, Smith RM. Morphological Classification of Grass Phytoliths. Soil Sci Soc Am J. 1969;33: 109–115. doi:10.2136/sssaj1969.03615995003300010030x

12. Ajaib M: Ethnobotanical Studies on Some Plants of... - Google Scholar. [cited 21 Mar 2021]. Available: https://scholar.google.co.in/scholar?hl=en&as_sdt=0%2C5&q=Ajaib+M%3A+Ethnobotanical+Studies+on+Some+Plants+of+District+Kotli%2C+Azad+Jammu+and+%09Kashmir.+MPhil+thesis.+GC+University%2C+Sustainable+Development+Study+Center%3B+2008.&btnG=

13. Ma JF, Miyake Y, Takahashi E. Chapter 2 Silicon as a beneficial element for crop plants. Studies in Plant Science. 2001. pp. 17–39. doi:10.1016/S0928-3420(01)80006-9

14. Sommer M, Kaczorek D, Kuzyakov Y, Breuer J. Silicon pools and fluxes in soils and landscapes - A review. Journal of Plant Nutrition and Soil Science. 2006. pp. 310–329. doi:10.1002/jpln.200521981

15. Sahebi M, Hanafi M, … ASNA-B research, 2015 undefined. Importance of silicon and mechanisms of biosilica formation in plants. hindawi.com. [cited 20 Mar 2021]. Available: https://www.hindawi.com/journals/bmri/2015/396010/abs/

16. Fraysse F, Pokrovsky OS, Schott J, Meunier JD. Surface properties, solubility and dissolution kinetics of bamboo phytoliths. Geochim Cosmochim Acta. 2006;70: 1939–1951. doi:10.1016/j.gca.2005.12.025

17. Tomlinson PB. Anatomy of the monocotyledons. III. Commelinales-Zingiberales. Anat monocotyledons III Commelinales-Zingiberales. 1969.

18. Bozarth SR. Classification of Opal Phytoliths Formed in Selected Dicotyledons Native to the Great Plains. Phytolith Systematics. Springer US; 1992. pp. 193–214. doi:10.1007/978-1-4899-1155-1_10

19. Rosen AM. Preliminary Identification of Silica Skeletons from Near Eastern Archaeological Sites: An Anatomical Approach. Phytolith Systematics. Springer US; 1992. pp. 129–147. doi:10.1007/978-1-4899-1155-1_7

20. Piperno D. Phytoliths: a comprehensive guide for archaeologists and paleoecologists. 2006. Available: https://books.google.co.in/books?hl=en&lr=&id=VWYnAAAAQBAJ&oi=fnd&pg=PP3&dq=Piperno,+D.R.,+2006.+Phytoliths:+a+comprehensive+guide+for+archaeologists+and+%09paleoecologists,+Lanham,+Maryland,+Alta+Mira+Press,+89−102&ots=uRIq9cozOx&sig=547RCVNm-B0pMUGijISL0OrfJro

21. Piperno DR, Pearsall DM, Washington DC. The Silica Bodies of Tropical American Grasses: Morphology, Taxonomy, and Implications for Grass Systematics and Fossil Phytolith Identification. repository.si.edu. Available: https://repository.si.edu/bitstream/handle/10088/7002/scb-0085.pdf

22. McKeague JA, Cline MG. SILICA IN SOIL SOLUTIONS: II. THE ADSORPTION OF MONOSILICIC ACID BY SOIL AND BY OTHER SUBSTANCES. Can J Soil Sci. 1963;43: 83–96. doi:10.4141/cjss63-011

23. Takahashi E, Ma J, and YM-C on A, 1990 undefined. The possibility of silicon as an essential element for higher plants. cabdirect.org. [cited 20 Mar 2021]. Available: https://www.cabdirect.org/cabdirect/abstract/19921964619

24. Ma Z, Liu X, Guan Y, A HL-C and S, 2006 undefined. Synthesis of magnetic silica nanospheres with metal ligands and application in affinity separation of proteins. Elsevier. [cited 20 Mar 2021]. Available: https://www.sciencedirect.com/science/article/pii/S0927775705006771?casa_token=V4IqnlQpwUQAAAAA:eV0hL9fE4_oAccLjkD7atJLCxro1Y0p-eQoH0V7Ki25qOdQFP9oEw9bJA2Er1ZPFd33Em7-jvQ

25. The method for determination of plant available silica... - Google Scholar. [cited 20 Mar 2021]. Available: https://scholar.google.co.in/scholar?hl=en&as_sdt=0%2C5&q=The+method+for+determination+of+plant+available+silica+in+soil.&btnG=

26. Copper distribution and speciation in bamboo exposed... - Google Scholar. [cited 20 Mar 2021]. Available: https://scholar.google.co.in/scholar?hl=en&as_sdt=0%2C5&q=+Copper+distribution+and+speciation+in+bamboo++exposed+to+a+high+Cu+concentration+and+Si+supplementation.+First+evidence+on+the&btnG=

27. Feng J, Shi Q, Wang X, Wei M, Yang F, Horticulturae HX-S, et al. Silicon supplementation ameliorated the inhibition of photosynthesis and nitrate metabolism by cadmium (Cd) toxicity in Cucumis sativus L. Elsevier. [cited 20 Mar 2021]. Available:

https://www.sciencedirect.com/science/article/pii/S030442380900483X?casa_token=sIFLScV8YPkAAAAA:lVSSVU_HWrUKLl99fyUtxsDxRepb3P06LCSbE4BB9IUcLDuyl7KcMSTmqpYTQWEJkKhWoyCdfg

28. Ma J, Takahashi E. Soil, fertilizer, and plant silicon research in Japan. 2002. Available: https://books.google.co.in/books?hl=en&lr=&id=gv420s5vm8QC&oi=fnd&pg=PP1&dq=Ma,+J.F.,+Takahashi,+E.,+2002.+Soil,+Fertiliser,+and+Plant+Silicon+Research+in+Japan.+Elsevier,+%09Amsterdam,+The+Netherlands.&ots=NfPsCxCMnO&sig=kzfDOvwOq95Tr6-1ewsclClaMc8

29. Epstein E. The anomaly of silicon in plant biology. Natl Acad Sci. 1994. Available: https://www.pnas.org/content/91/1/11.short

30. Vicari M, evolution DB-T in ecology &, 1993 undefined. Do grasses fight back? The case for antiherbivore defences. Elsevier. [cited 20 Mar 2021]. Available: https://www.sciencedirect.com/science/article/pii/016953479390026L

31. Massey FP, Roland Ennos A, Hartley SE. Herbivore specific induction of silica-based plant defences. Oecologia. 2007;152: 677–683. doi:10.1007/s00442-007-0703-5

32. Dabney C, Ostergaard J, Watkins E, Chen C. A novel method to characterize silica bodies in grasses. Plant Methods. 2016;12. doi:10.1186/s13007-016-0108-8

33. Chauhan D, Tripathi D, … DK-IJIBC, 2011 undefined. Diversity, distribution and frequency based attributes of phytolith in Arundo donax L. academia.edu. [cited 20 Mar 2021]. Available: https://www.academia.edu/download/14985339/ijibcs-1012-039_t.pdf

34. Bartoli F, Journal LW-SSS of A, 1980 undefined. Dissolution of biogenic opal as a function of its physical and chemical properties. Wiley Online Libr. [cited 20 Mar 2021]. Available: https://acsess.onlinelibrary.wiley.com/doi/abs/10.2136/sssaj1980.03615995004400040043x

35. Li Z, Song Z, Cornelis JT. Impact of rice cultivar and organ on elemental composition of phytoliths and the release of bio-available silicon. Front Plant Sci. 2014;5. doi:10.3389/fpls.2014.00529

36. Zhang X, Song Z, Hao Q, Wang Y, Ding F, Song A. Phytolith-occluded carbon storages in forest litter layers in southern china: Implications for evaluation of long-term forest carbon budget. Front Plant Sci. 2019;10. doi:10.3389/fpls.2019.00581

37. Smithson F. Plant opal in soil [29]. Nature. 1956. p. 107. doi:10.1038/178107a0

38. Alexandre A, Meunier J-D, Colin F, Koud J-M, Koud' J-M. Plant impact on the biogeochemical cycle of silicon and related weathering processes LETTER Plant impact on the biogeochemical cycle of silicon and related weathering processes. Geochim Cosmochim Acta. 1997;61: 677–682. doi:10.1016/S0016-7037(97)00001-Xï

39. Shakoor S, Bhat M, Mir S, Sci AS-UJP, 2014 undefined. Investigations into phytoliths as diagnostic markers for the grasses (Poaceae) of Punjab. academia.edu. [cited 20 Mar 2021]. Available: https://www.academia.edu/download/49594441/UJPS2-10302265.pdf

40. Gramineae. CM-A of the monocotyledons. 1., 1960 undefined. Anatomy of the monocotyledons. 1. Gramineae. cabdirect.org. [cited 20 Mar 2021]. Available: https://www.cabdirect.org/cabdirect/abstract/19610700925

41. Hodson MJ. The relative importance of cell wall and lumen phytoliths in carbon sequestration in soil: A hypothesis. Front Earth Sci. 2019;7. doi:10.3389/feart.2019.00167

42. Shillito LM. Grains of truth or transparent blindfolds? A review of current debates in archaeological phytolith analysis. Vegetation History and Archaeobotany. Springer Science and Business Media, LLC; 2013. pp. 71–82. doi:10.1007/s00334-011-0341-z

43. Carnelli AL, Theurillat JP, Madella M. Phytolith types and type-frequencies in subalpine-alpine plant species of the European Alps. Rev Palaeobot Palynol. 2004;129: 39–65. doi:10.1016/j.revpalbo.2003.11.002

44. Hodson MJ. The development of phytoliths in plants and its influence on their chemistry and isotopic composition. Implications for palaeoecology and archaeology. J Archaeol Sci. 2016;68: 62–69. doi:10.1016/j.jas.2015.09.002

45. Zuo X, Lu H. Phytolith Radiocarbon Dating: A Review of Previous Studies in China and the Current State of the Debate. Front Plant Sci. 2019;10. doi:10.3389/fpls.2019.01302

46. Botany MH-A of, 1986 undefined. Silicon Deposition in the Roots, Culm and Leaf of Phalaris canariensis L. academic.oup.com. [cited 23 Mar 2021]. Available: https://academic.oup.com/aob/article-abstract/58/2/167/137698

47. Hodson M, Microscopy AS-S, 1990 undefined. Techniques for the microanalysis of higher plants with particular reference to silicon in cryofixed wheat tissues. cabdirect.org. [cited 23 Mar 2021]. Available: https://www.cabdirect.org/cabdirect/abstract/19910743656

48. Hodson M, White P, Mead A, botany MB-A of, 2005 undefined. Phylogenetic variation in the silicon composition of plants. academic.oup.com. [cited 23 Mar 2021]. Available: https://academic.oup.com/aob/article-abstract/96/6/1027/216512

49. Carnelli AL, Madella{ M. Biogenic Silica Production in Selected Alpine Plant Species and Plant Communities. academic.oup.com. 2001;87: 425–434. doi:10.1006/anbo.2000.1355

50. Song Z, McGrouther K, Reviews HW-E-S, 2016 undefined. Occurrence, turnover and carbon sequestration potential of phytoliths in terrestrial ecosystems. Elsevier. [cited

23 Mar 2021]. Available: https://www.sciencedirect.com/science/article/pii/S0012825216300733

51. Zurro D, García-Granero JJ, Lancelotti C, Madella M. Directions in current and future phytolith research. J Archaeol Sci. 2016;68: 112–117. doi:10.1016/j.jas.2015.11.014

52. Epstein: Annual Review of Plant Physiollogy and Plant... - Google Scholar. [cited 20 Mar 2021]. Available: https://scholar.google.co.in/scholar?q=related:NNkU_e8jh1YJ:scholar.google.com/&scioq=Epstein,+E.+(1999).+Annual+Review+of+Plant+Physiology+and+Plant+Molecular+Biology.+50:+%09641–644p.&hl=en&as_sdt=0,5

53. Ma JF, Yamaji N. Silicon uptake and accumulation in higher plants. Trends in Plant Science. 2006. pp. 392–397. doi:10.1016/j.tplants.2006.06.007

54. Matoh T, Kairusmee P, Takahashi E. Salt-Induced Damage to Rice Plants and Alleviation Effect of Silicate. Soil Sci Plant Nutr. 1986;32: 295–304. doi:10.1080/00380768.1986.10557506

55. Bhat MA, Shakoor SA, Badgal P, Soodan AS. Taxonomic demarcation of setaria pumila (Poir.) Roem. & Schult., S. verticillata (L.) P. Beauv., and S. Viridis (L.) P. Beauv. (cenchrinae, paniceae, panicoideae, poaceae) from phytolith signatures. Front Plant Sci. 2018;9. doi:10.3389/fpls.2018.00864

56. Bremond L, Alexandre A, Véla E, Guiot J. Advantages and disadvantages of phytolith analysis for the reconstruction of Mediterranean vegetation: An assessment based on modern phytolith, pollen and botanical data (Luberon, France). Rev Palaeobot Palynol. 2004;129: 213–228. doi:10.1016/j.revpalbo.2004.02.002

57. Bremond L, Alexandre A, Wooller MJ, Hély C, Williamson D, Schäfer PA, et al. Phytolith indices as proxies of grass subfamilies on East African tropical mountains. Glob Planet Change. 2008;61: 209–224. doi:10.1016/j.gloplacha.2007.08.016

58. Barboni D, Bremond L, Palaeogeography RB-, Palaeoclimatology undefined, 2007 undefined. Comparative study of modern phytolith assemblages from inter-tropical Africa. Elsevier. [cited 23 Mar 2021]. Available: https://www.sciencedirect.com/science/article/pii/S003101820600602X

59. Micropaleontology JC-, 1999 undefined. Late devonian, permian and triassic phytoliths from antarctica. pubs.geoscienceworld.org. [cited 20 Mar 2021]. Available: https://pubs.geoscienceworld.org/micropress/micropal/article/45/1/56/85077

60. Fredlund GG, Tieszen LT. Modern Phytolith Assemblages from the North American Great Plains. J Biogeogr. 1994;21: 321. doi:10.2307/2845533

61. Morcote-Ríos G, Bernal R, Raz L. Phytoliths as a tool for archaeobotanical, palaeobotanical and palaeoecological studies in Amazonian palms. Bot J Linn Soc. 2016;182: 348–360. doi:10.1111/boj.12438

62. Silantyeva M, Solomonova M, Speranskaja N, Blinnikov MS. Phytoliths of temperate forest-steppe: A case study from the Altay, Russia. Rev Palaeobot Palynol. 2018;250: 1–15. doi:10.1016/j.revpalbo.2017.12.002

63. Collura L, International KN-Q, 2017 undefined. Wood and bark phytoliths of West African woody plants. Elsevier. [cited 23 Mar 2021]. Available: https://www.sciencedirect.com/science/article/pii/S1040618215014652?casa_token=ZCaaBeVAlMoAAAAA:YcSBjz9O5_YDypS-N0_YxQjX77CoXdHG0R595s9mEkXq3-5MXvQd4ISueh269SErsn_GTg1fNQ

64. Contreras S, International AZ-Q, 2019 undefined. Late Quaternary vegetation history based on phytolith records in the eastern Chaco (Argentina). Elsevier. [cited 20 Mar 2021]. Available: https://www.sciencedirect.com/science/article/pii/S1040618217311412?casa_token=IxQPGKxK0eoAAAAA:6-C0cv1F2dYjC_dueas3CugmhIf-0u0A2atwez3oHxcCCMOARms__LxhAEaQYMtpj5aKb_WZSw

65. Danu M, Messager E, Carozza JM, Carozza L, Bouby L, Philibert S, et al. Phytolith evidence of cereal processing in the Danube Delta during the Chalcolithic period. Quat Int. 2019;504: 128–138. doi:10.1016/j.quaint.2018.03.033

66. Lux A, Lukačová Z, Vaculík M, Švubová R, Kohanová J, Soukup M, et al. Silicification of Root Tissues. Plants. 2020;9: 111. doi:10.3390/plants9010111

67. Li Z, de Tombeur F, Linden C Vander, Cornelis JT, Delvaux B. Soil microaggregates store phytoliths in a sandy loam. Geoderma. 2020;360: 114037. doi:10.1016/j.geoderma.2019.114037

68. Piperno D, International CM-Q, 2020 undefined. Phytoliths in modern plants from amazonia and the neotropics at large: Implications for vegetation history reconstruction. Elsevier. [cited 20 Mar 2021]. Available: https://www.sciencedirect.com/science/article/pii/S1040618220306698?casa_token=E9hASoRhfIsAAAAA:Hfx1-L7e_SqlknN0dB4lWMowL0OiG_vzGAbz7IjZ0xB2-G2Ul8F3jIwAM7TjKc6QkEtMwLRlGg

69. Testé M, Garnier A, Limondin-Lozouet N, Oxlaj E, Castanet C, Purdue L, et al. The phytoliths of Naachtun (Petén, Guatemala): Development of a modern reference for the characterization of plant communities in the Maya Tropical Lowlands. Rev Palaeobot Palynol. 2020;272: 104130. doi:10.1016/j.revpalbo.2019.104130

70. Gao G, Jie D, Li D, Li N, Liu L, Liu H, et al. Reliability of phytoliths for reconstructing vegetation dynamics in northern temperate forest regions: A case study in northeast China. Elsevier. [cited 20 Mar 2021]. Available: https://www.sciencedirect.com/science/article/pii/S027737911830266X

71. Science SM-J of A, 1989 undefined. Phytolith shape frequencies in North Dakota grasses: a comparison to general patterns. Elsevier. [cited 20 Mar 2021]. Available:

https://www.sciencedirect.com/science/article/pii/0305440389900708

72. Mulholland SC, Rapp G. A Morphological Classification of Grass Silica-Bodies. Phytolith Systematics. Springer US; 1992. pp. 65–89. doi:10.1007/978-1-4899-1155-1_4

73. Ghosh R, Naskar M, Bera S. Phytolith assemblages of grasses from the Sunderbans, India and their implications for the reconstruction of deltaic environments. Palaeogeogr Palaeoclimatol Palaeoecol. 2011;311: 93–102. doi:10.1016/j.palaeo.2011.08.009

74. Yost CL, Jackson LJ, Stone JR, Cohen AS. Subdecadal phytolith and charcoal records from Lake Malawi, East Africa imply minimal effects on human evolution from the ∼74 ka Toba supereruption. J Hum Evol. 2018;116: 75–94. doi:10.1016/j.jhevol.2017.11.005

75. technology JC-S, 1960 undefined. Preparation of leaf epidermis for topographic study. Taylor Fr. [cited 20 Mar 2021]. Available: https://www.tandfonline.com/doi/abs/10.3109/10520296009114713

76. Madella M, Alexandre A, Ball T. International code for phytolith nomenclature 1.0. Ann Bot. 2005;96: 253–260. doi:10.1093/aob/mci172

77. Neumann K, Strömberg CAE, Ball T, Albert RM, Vrydaghs L, Cummings LS. International Code for Phytolith Nomenclature (ICPN) 2.0. Ann Bot. 2019;124: 189–199. doi:10.1093/aob/mcz064

78. Ball T, Davis A, Evett R, Ladwig J, … MT-J of, 2016 undefined. Morphometric analysis of phytoliths: recommendations towards standardization from the International Committee for Phytolith Morphometrics. Elsevier. [cited 20 Mar 2021]. Available: https://www.sciencedirect.com/science/article/pii/S0305440315001168?casa_token=5acnBAjl7UkAAAAA:HBavzmRAgd3gSrlosa4CDnvt7Bm_3jYjBZ9yChm95r6cDBkY3uDXvdoYCA8J_HCgJUQcwtaLJA

79. Lentfer CJ, Boyd WE. A comparison of three methods for the extraction of phytoliths from sediments. J Archaeol Sci. 1998;25: 1159–1183. doi:10.1006/jasc.1998.0286

80. Parr JF, Dolic V, Lancaster G, Boyd WE. A microwave digestion method for the extraction of phytoliths from herbarium specimens. Rev Palaeobot Palynol. 2001;116: 203–212. doi:10.1016/S0034-6667(01)00089-6

81. Palynology JP-R of P and, 2002 undefined. A comparison of heavy liquid floatation and microwave digestion techniques for the extraction of fossil phytoliths from sediments. Elsevier. [cited 23 Mar 2021]. Available: https://www.sciencedirect.com/science/article/pii/S0034666701001385

82. Jenkins E, Jamjoum K, Nuimat S, … RS-J of, 2019 undefined. Corrigendum to

"Identifying ancient water availability through phytolith analysis: An experimental approach" J. Archaeol. Sci. 73 (2016) 82–93. core.ac.uk. [cited 23 Mar 2021]. Available: https://core.ac.uk/download/pdf/326517807.pdf

83. Jaggi N, Spectroscopy DV-H of ASS, 2006 undefined. FOURIER TRANSFORM INFRARED SPECTROSCOPY. Springer. 2007; 411–450. doi:10.1007/0-387-37590-2_9

84. Clayton WD, Vorontsova MS, Harman KT, Williamson H. GrassBase - The Online World Grass Flora. GrassBase - Online World Grass Flora. 2016.

85. Barker NP, Clark LG, Davis JI, Duvall MR, Guala GF, Hsiao C, et al. Phylogeny and subfamilial classification of the grasses (Poaceae). Ann Missouri Bot Gard. 2001;88: 373–457. doi:10.2307/3298585

86. Aliscioni S, Bell HL, Besnard G, Christin PA, Columbus JT, Duvall MR, et al. New grass phylogeny resolves deep evolutionary relationships and discovers C 4 origins. New Phytol. 2012;193: 304–312. doi:10.1111/j.1469-8137.2011.03972.x

87. Lanning FC, Eleuterius LN. Silica and ash in native plants of the central and southeastern regions of the United States. Ann Bot. 1987;60: 361–375. doi:10.1093/oxfordjournals.aob.a087456

88. Lanning FC, Eleuterius LN. Silica deposition in some C3 and C4 species of grasses, sedges and composites in the USA. Ann Bot. 1989;64: 395–410. doi:10.1093/oxfordjournals.aob.a087858

89. Mukherjee B, Biswas O, Ghosh R, Paruya DK, Bera S. Phytolith spectra of some eastern Himalayan pteridophytes: Assessing potential for distinguishing different vegetation–climate zones along the elevation gradient. Flora Morphol Distrib Funct Ecol Plants. 2019;254: 95–112. doi:10.1016/j.flora.2018.08.013

90. Srivastava A. 1978. Study of leaf epidermis in the... - Google Scholar. [cited 20 Mar 2021]. Available: https://scholar.google.co.in/scholar?hl=en&as_sdt=0%2C5&q=Srivastava+A.+1978.+Study+of+leaf+epidermis+in+the+genus+Digitaria+Rich.+%28Gramineae%29.+J+%09Indian+Bot+Soc.+37%3A155–160&btnG=

91. Stace C. 1984. The taxonomic importance of the leaf... - Google Scholar. [cited 20 Mar 2021]. Available: https://scholar.google.co.in/scholar?hl=en&as_sdt=0%2C5&q=Stace+C.+1984.+The+taxonomic+importance+of+the+leaf+surface.+In%3A+Herwood+VH%2C+Moore+DM%2C+%09editors.+Current+Concepts+in+Plant+Taxonomy%2C+Systematic+Association%2C+vol.+25.+%09London+and+Orlando%3A+Academic+Press%3B+p.+628–642&btnG=

92. Folorunso A, Oyetunji O. Comparative Foliar Epidermal Studies in Cymbopogon Citratus (Stapf.) and Cymbopogon Giganteus (Hochst.) Chiov. in Nigeria. 2007 [cited

23 Mar 2021]. Available: http://ir.oauife.edu.ng/handle/123456789/3383

93. Desai RJ, Raole VM, Arya A. Comparative Foliar Epidermal Studies in Coix lacryma-jobi L. and Coix aquatica Roxb. (Poaceae). Not Sci Biol. 2009;1: 37–40. Available: www.notulaebiologicae.ro

94. Alli A. Comparative vegetative and foliar epidermal features of three Paspalum L. species in Edo State, Nigeria Biodiversity and conservation of leafy vegetables in Edo State, Nigeria View project Plant Anatomy View project Ogie-Odia Efosa. researchgate.net. 2010. Available: https://www.researchgate.net/publication/230868584

95. Ahmad F, Khan M, Ahmad M. Foliar epidermal anatomy as an aid to the identification of grasses in tribe Aveneae (subfamily Pooideae, Poaceae) from salt range of Pakistan Male contraceptives View project Medicinal Plants and Ethno-pharmacology View project. Artic J Med Plant Res. 2011. Available: https://www.researchgate.net/publication/261633937

96. Shaheen S, Ahmad M, Khan F, … MZ-J of M, 2011 undefined. Micormorphological diversity in leaf epidermal anatomy of Brachiaria species using elemental dispersive spectrophotometer analysis. lcwu.edu.pk. [cited 20 Mar 2021]. Available: http://www.lcwu.edu.pk/ocd/cfiles/Botany/BOT-735/02.BrachiariaEDS-September2011.pdf

97. systematics PT-P, 1992 undefined. Predicted World Distribution of C3 and C4 Grass Phytoliths. Springer. [cited 20 Mar 2021]. Available: https://link.springer.com/chapter/10.1007/978-1-4899-1155-1_6

98. Shakoor S, Bhat M, Flora AS-, 2016 undefined. Taxonomic demarcation of Arundo donax L. and Phragmites karka (Retz.) Trin. ex Steud.(Arundinoideae, Poaceae) from phytolith signatures. Elsevier. [cited 20 Mar 2021]. Available: https://www.sciencedirect.com/science/article/pii/S036725301630113X?casa_token=TNpvbc34H4YAAAAA:UguwnVE9hsPS-2p-Mv71Qb1UxJ3DCYeacJ615e_0v2fRa3tRlalccI-cpkC0TosY8iLmPV_GiQ

99. Out WA, Madella M. Towards improved detection and identification of crop by-products: Morphometric analysis of bilobate leaf phytoliths of Pennisetum glaucum and Sorghum bicolor. Quat Int. 2017;434: 1–14. doi:10.1016/j.quaint.2015.07.017

100. Gallaher TJ, Akbar SZ, Klahs PC, Marvet CR, Senske AM, Clark LG, et al. 3D shape analysis of grass silica short cell phytoliths: a new method for fossil classification and analysis of shape evolution. New Phytol. 2020;228: 376–392. doi:10.1111/nph.16677

101. Lima J, Leite K, Clark L, PhytoKeys RO-, 2021 undefined. Notes on leaf micromorphology of the rare herbaceous bamboo Buergersiochloa bambusoides Pilg.(Olyreae, Poaceae) from New Guinea and its taxonomic. ncbi.nlm.nih.gov. [cited 20 Mar 2021]. Available: https://www.ncbi.nlm.nih.gov/pmc/articles/pmc7910262/

102. Lima JF, Leite KRB, Clark LG, De Oliveira RP. Leaf micromorphology in Poaceae subtribe Olyrinae (Bambusoideae) and its systematic implications. Bot J Linn Soc. 2020;192: 184–207. doi:10.1093/botlinnean/boz071

103. Parry D, … MH-… T of the, 1984 undefined. Some recent advances in studies of silicon in higher plants. royalsocietypublishing.org. [cited 20 Mar 2021]. Available: https://royalsocietypublishing.org/doi/abs/10.1098/rstb.1984.0045

104. Hodson M, Botany AS-A of, 1988 undefined. Observations on the Distribution of Mineral Elements in the Leaf of Wheat (Triticum aestivum L.), with Particular Reference to Silicon. academic.oup.com. [cited 20 Mar 2021]. Available: https://academic.oup.com/aob/article-abstract/62/5/463/204880

105. Bonnett, O. T. (1972). Silicificd Cells of Grasses:... - Google Scholar. [cited 23 Mar 2021]. Available: https://scholar.google.co.in/scholar?hl=en&as_sdt=0%2C5&q=Bonnett%2C+O.+T.+%281972%29.+Silicificd+Cells+of+Grasses%3A+A+Major+Source+of+Plant+Opal+in+Illinois+%09Soils.+New+York%2C+NY%3A+University+of+lllinois+at+Urbana-Champaign%2C+College+of+%09Agriculture.&btnG=

106. Ball TB, Gardner JS, Anderson N. Identifying inflorescence phytoliths from selected species of wheat (*Triticum monococcum, T. dicoccon, T. dicoccoides*, and *T. aestivum*) and barley (*Hordeum vulgare* and *H. spontaneum*) (Gramineae). Am J Bot. 1999;86: 1615–1623. doi:10.2307/2656798

107. Lu H, Liu KB. Phytoliths of common grasses in the coastal environments of southeastern USA. Estuar Coast Shelf Sci. 2003;58: 587–600. doi:10.1016/S0272-7714(03)00137-9

108. Ball TB, Ehlers R, Standing MD. Review of typologic and morphometric analysis of phytoliths produced by wheat and barley. Breed Sci. 2009. Available: https://www.jstage.jst.go.jp/article/jsbbs/59/5/59_5_505/_article/-char/ja/

109. Tripathi DK, Kumar R, Pathak AK, Chauhan DK, Rai AK. Laser-Induced Breakdown Spectroscopy and Phytolith Analysis: An Approach to Study the Deposition and Distribution Pattern of Silicon in Different Parts of Wheat (Triticum aestivum L.) Plant. Agric Res. 2012;1: 352–361. doi:10.1007/s40003-012-0042-6

110. Bot TJ, Lisztes-Szabó Z, Kovács S, Pető Á. Turkish Journal of Botany Phytolith analysis of Poa pratensis (Poaceae) leaves. journals.tubitak.gov.tr. [cited 20 Mar 2021]. doi:10.3906/bot-1311-8

111. Jattisha PI, Sabu M. Foliar phytoliths as an aid to the identification of Paniceae (Panicoideae: Poaceae) grasses in South India. Webbia. 2015;70: 115–131. doi:10.1080/00837792.2015.1005908

112. Naskar M, Bera S. Taxonomic assessment of opal phytoliths from grasses of deltaic West Bengal, India. Nord J Bot. 2018;36. doi:10.1111/njb.01695

113. Murungi M, Palynology MB-R of P and, 2020 undefined. Revised taxonomic interpretations of Cyperaceae phytoliths for (paleo) botanical studies with some notes on terminology. Elsevier. [cited 20 Mar 2021]. Available: https://www.sciencedirect.com/science/article/pii/S0034666719301174

114. Ge Y, Lu H, Wang C, reports XG-S, 2020 undefined. Phytoliths in selected broad-leaved trees in China. nature.com. [cited 20 Mar 2021]. Available: https://www.nature.com/articles/s41598-020-72547-w

115. Watling J, Castro MT, Simon MF, Rodrigues FO, Brilhante de Medeiros M, De Oliveira PE, et al. Phytoliths from native plants and surface soils from the Upper Madeira river, SW Amazonia, and their potential for paleoecological reconstruction. Quat Int. 2020;550: 85–110. doi:10.1016/j.quaint.2020.03.045

116. Lu H, Zhang J, Wu N, Liu K, Xu D, Li Q. Phytoliths Analysis for the Discrimination of Foxtail Millet (Setaria italica) and Common Millet (Panicum miliaceum). Bendahmane M, editor. PLoS One. 2009;4: e4448. doi:10.1371/journal.pone.0004448

117. Luo W, Yang Y, Zhuang L, Gan · Huiyuan, Chunguang Gu ·, Huang C, et al. Phytolith evidence of water management for rice growing and processing between 8,500 and 7,500 cal years bp in the middle Huai river valley, China. Veg Hist Archaeobot. 2021;30: 243–254. doi:10.1007/s00334-020-00782-2

118. Ge Y, Lu H, Zhang J, Wang C, Gao X. Phytoliths in Inflorescence Bracts: Preliminary Results of an Investigation on Common Panicoideae Plants in China. Front Plant Sci. 2020;10: 1736. doi:10.3389/fpls.2019.01736

119. Tripathi DK, Mishra S, Chauhan DK, Tiwari SP, Kumar C. Typological and frequency based study of opaline silica (Phytolith) Deposition in two common Indian sorghum L. species. Proc Natl Acad Sci India Sect B - Biol Sci. 2013;83: 97–104. doi:10.1007/s40011-012-0066-5

120. KRISHNAN S, SAMSON NP, RAVICHANDRAN P, NARASIMHAN D, DAYANANDAN P. Phytoliths of Indian grasses and their potential use in identification. Bot J Linn Soc. 2000;132: 241–252. doi:10.1111/j.1095-8339.2000.tb01529.x

121. Watling K, Parr J, Rintoul L, … CB-SAP, 2011 undefined. Raman, infrared and XPS study of bamboo phytoliths after chemical digestion. Elsevier. [cited 20 Mar 2021]. Available: https://www.sciencedirect.com/science/article/pii/S1386142511001521?casa_token=xboB06epB0UAAAAA:Jd86_R6jn9owfIhnYya9eJ2WB44XBIyxY2INcUURzqo1QjjnXYdgFsRyF4MdG74xwr6g1cdPYA

122. Corbineau R, Reyerson PE, Alexandre A, Santos GM. Towards producing pure phytolith concentrates from plants that are suitable for carbon isotopic analysis. Rev Palaeobot Palynol. 2013;197: 179–185. doi:10.1016/j.revpalbo.2013.06.001

123. Alexandre A, Basile-Doelsch I, Delhaye T, Borshneck D, Mazur JC, Reyerson P, et al. New highlights of phytolith structure and occluded carbon location: 3-D X-ray microscopy and NanoSIMS results. Biogeosciences. 2015;12: 863–873. doi:10.5194/bg-12-863-2015

124. Bertoluzza A, Fagnano C, Antonietta Morelli M, Gottardi V, Guglielmi M. Raman and infrared spectra on silica gel evolving toward glass. J Non Cryst Solids. 1982;48: 117–128. doi:10.1016/0022-3093(82)90250-2

125. Gopal NO, Narasimhulu K V., Rao JL. EPR, optical, infrared and Raman spectral studies of Actinolite mineral. Spectrochim Acta - Part A Mol Biomol Spectrosc. 2004;60: 2441–2448. doi:10.1016/j.saa.2003.12.021

126. Sol-Gel Science: The Physics and Chemistry of Sol-Gel Processing - C. Jeffrey Brinker, George W. Scherer - Google Books. [cited 23 Mar 2021]. Available: https://books.google.co.in/books?hl=en&lr=&id=CND1BAAAQBAJ&oi=fnd&pg=PP1&dq=Brinker,+C.+J.,+and+Scherer,+G.,W(1990).+Sol-Gel+Science:+The+Chemistry+and+Physics+of+Sol-%09Gel+Processing.+London:+Academic+Press&ots=aftNH9UkeC&sig=qhz50R18ZSzgBzHuOR_ccXx1MCc#v=onepage&q=Brinker%2C C. J.%2C and Scherer%2C G.%2CW(1990). Sol-Gel Science%3A The Chemistry and Physics of Sol-%09Gel Processing. London%3A Academic Press&f=false

127. Kow KW, Yusoff R, Aziz ARA, Abdullah EC. Characterisation of bio-silica synthesised from cogon grass (Imperata cylindrica). Powder Technol. 2014;254: 206–213. doi:10.1016/j.powtec.2014.01.018

128. Mourhly A, Khachani M, El Hamidi A, Kacimi M, Halim M, Arsalane S. The synthesis and characterization of low-cost mesoporous silica SiO2 from local pumice rock. Nanomater Nanotechnol. 2015;5. doi:10.5772/62033

129. Bansal V, Ahmad A, Chemical MS-J of the A, 2006 undefined. Fungus-mediated biotransformation of amorphous silica in rice husk to nanocrystalline silica. ACS Publ. [cited 20 Mar 2021]. Available: https://pubs.acs.org/doi/full/10.1021/ja062113+

130. Karunakaran G, Suriyaprabha R, Manivasakan P, Yuvakkumar R, Rajendran V, Prabu P, et al. Effect of nanosilica and silicon sources on plant growth promoting rhizobacteria, soil nutrients and maize seed germination. IET Nanobiotechnology. 2013;7: 70–77. doi:10.1049/iet-nbt.2012.0048

131. Socrates G. Infrared and Raman characteristic group frequencies: tables and charts. 2004. Available: https://books.google.co.in/books?hl=en&lr=&id=LDoAAjMnwEIC&oi=fnd&pg=PP21&dq=Socrates,+G.+(2001).+Infrared+and+Raman+Characteristic+Group+Frequencies:+Tables+and+%09Charts.+Chichester:+JohnWiley+and+Sons.&ots=Cq00UYFqbE&sig=0mrVVlPJO39n0n6qqag2L3MQ1Kg

132. Holm JL, Kleppa OJ, Westrum EF. Thermodynamics of polymorphic transformations in silica. Thermal properties from 5 to 1070° K and pressure-temperature stability fields for coesite and stishovite. Geochim Cosmochim Acta. 1967;31: 2289–2307. doi:10.1016/0016-7037(67)90003-8

133. Zhang J, Lu H, Wu N, Yang X, Diao X. Phytolith analysis for differentiating between foxtail millet (setaria italica) and green foxtail (setaria viridis). PLoS One. 2011;6. doi:10.1371/journal.pone.0019726

134. Lisztes-Szabó Z, Kovács S, … PB-AS, 2015 undefined. Quantifiable differences between phytolith assemblages detected at species level: analysis of the leaves of nine Poa species (Poaceae). yadda.icm.edu.pl. [cited 20 Mar 2021]. Available: http://yadda.icm.edu.pl/yadda/element/bwmeta1.element.agro-23ce40ed-c5e0-455b-add1-a63411a8705d

135. Espíndola-Gonzalez A, Martínez-Hernández AL, Angeles-Chávez C, Castaño VM, Velasco-Santos C. Novel crystalline SiO 2 nanoparticles via annelids bioprocessing of agro-industrial wastes. Nanoscale Res Lett. 2010;5: 1408–1417. doi:10.1007/s11671-010-9654-6

136. Espíndola-Gonzalez A, … RF-R-A in M, 2014 undefined. Structural characterization of silica particles extracted from grass Stenotaphrum secundatum: biotransformation via annelids. hindawi.com. [cited 20 Mar 2021]. Available: https://www.hindawi.com/journals/amse/2014/956945/abs/

137. Morris R, Vaniman D, … DB-P of the, 2016 undefined. Silicic volcanism on Mars evidenced by tridymite in high-SiO2 sedimentary rock at Gale crater. Natl Acad Sci. [cited 20 Mar 2021]. Available: https://www.pnas.org/content/113/26/7071.short

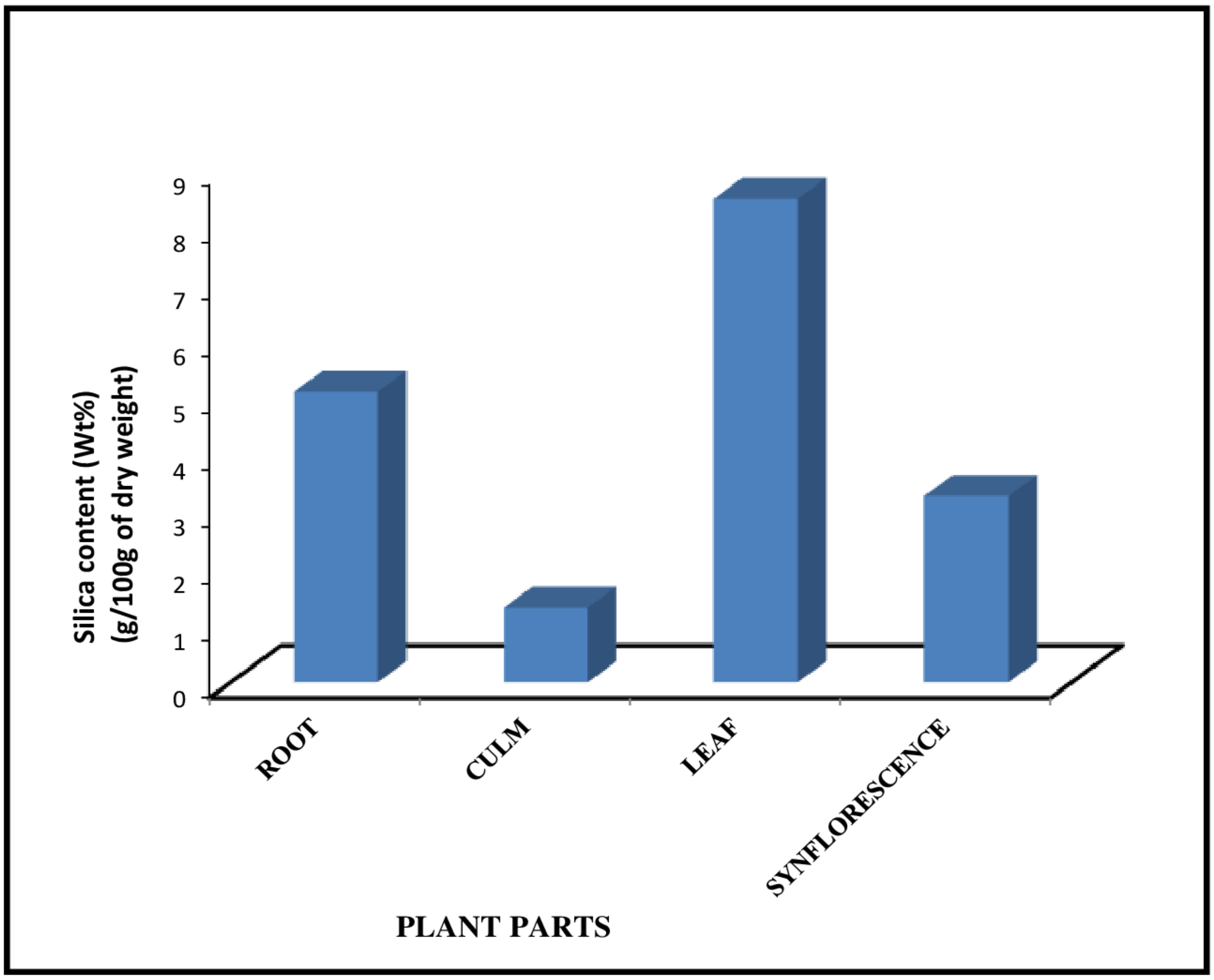
Silica content (Wt%)
(g/100g of dry weight)
0
1
2
3
4
5
6
7
8
9
ROOT
CULM
LEAF
SYNFLORESCENCE
PLANT PARTS

Fig. 1

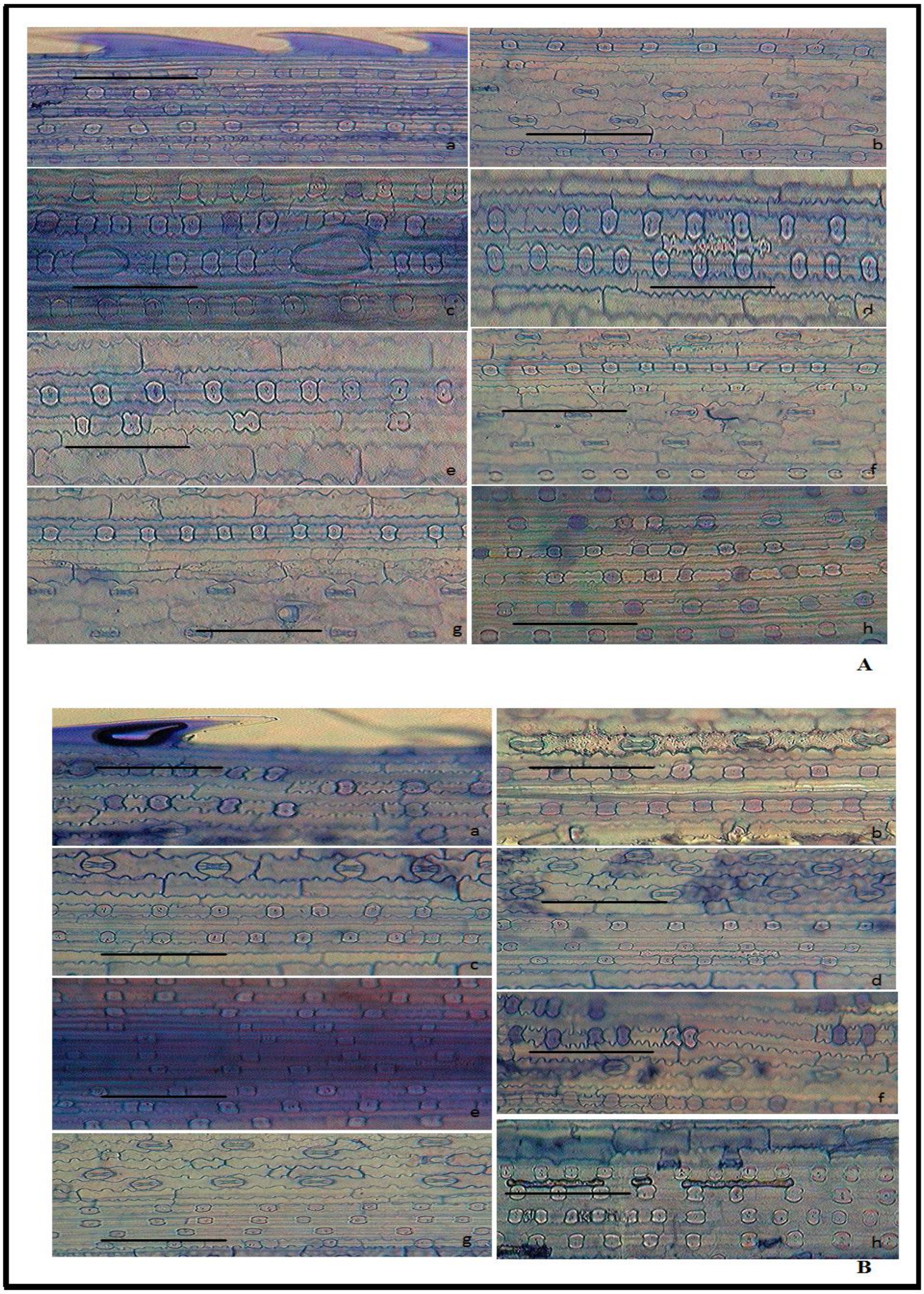

Fig. 2.

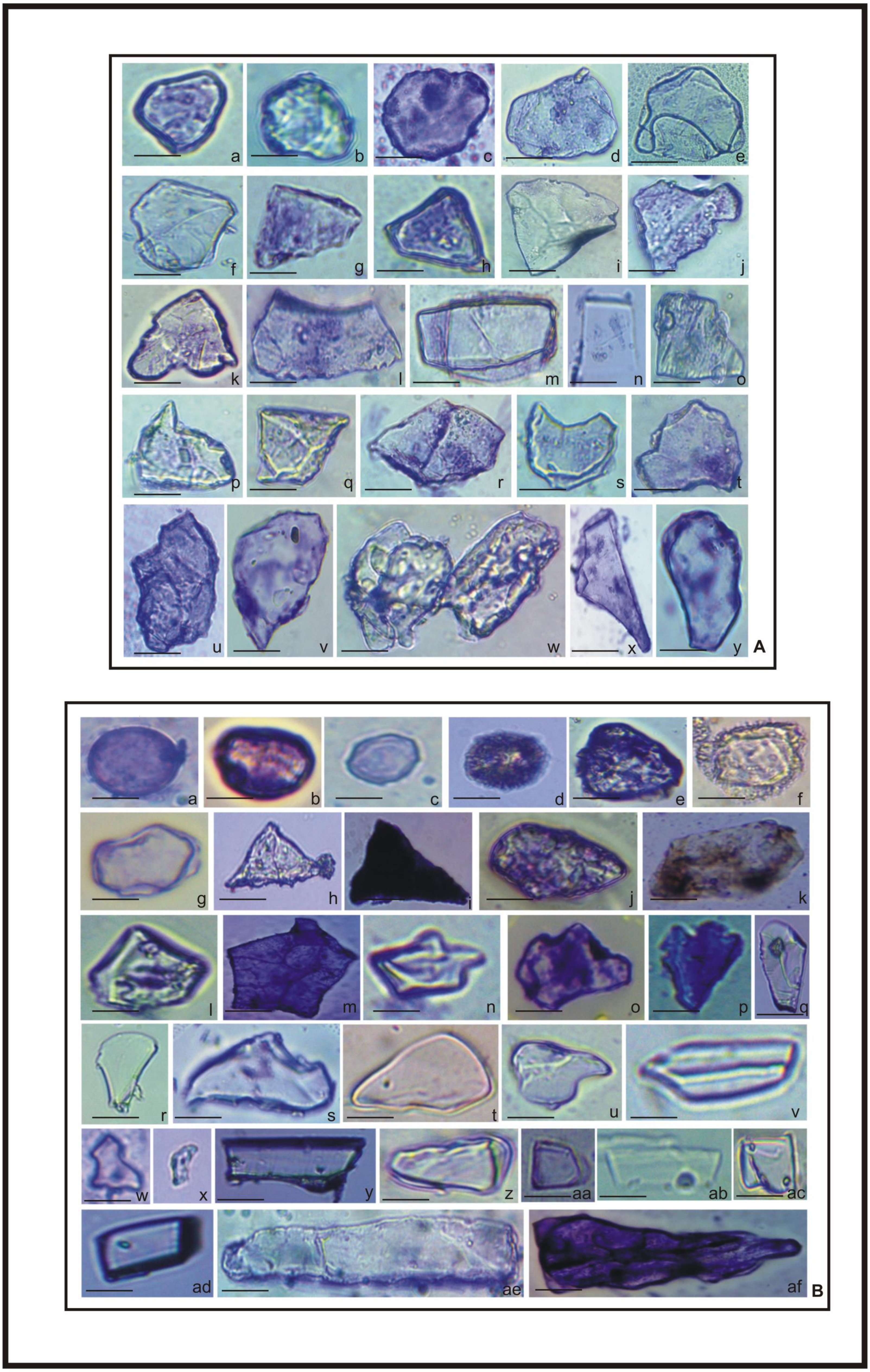
a b c d e
f g h i j
k l m n o
p q r s t
u v w x y
A
a b c d e f
g h i j k
l m n o p q
r s t u v
w x y z aa ab ac
ad ae af
B

Fig. 3A and B

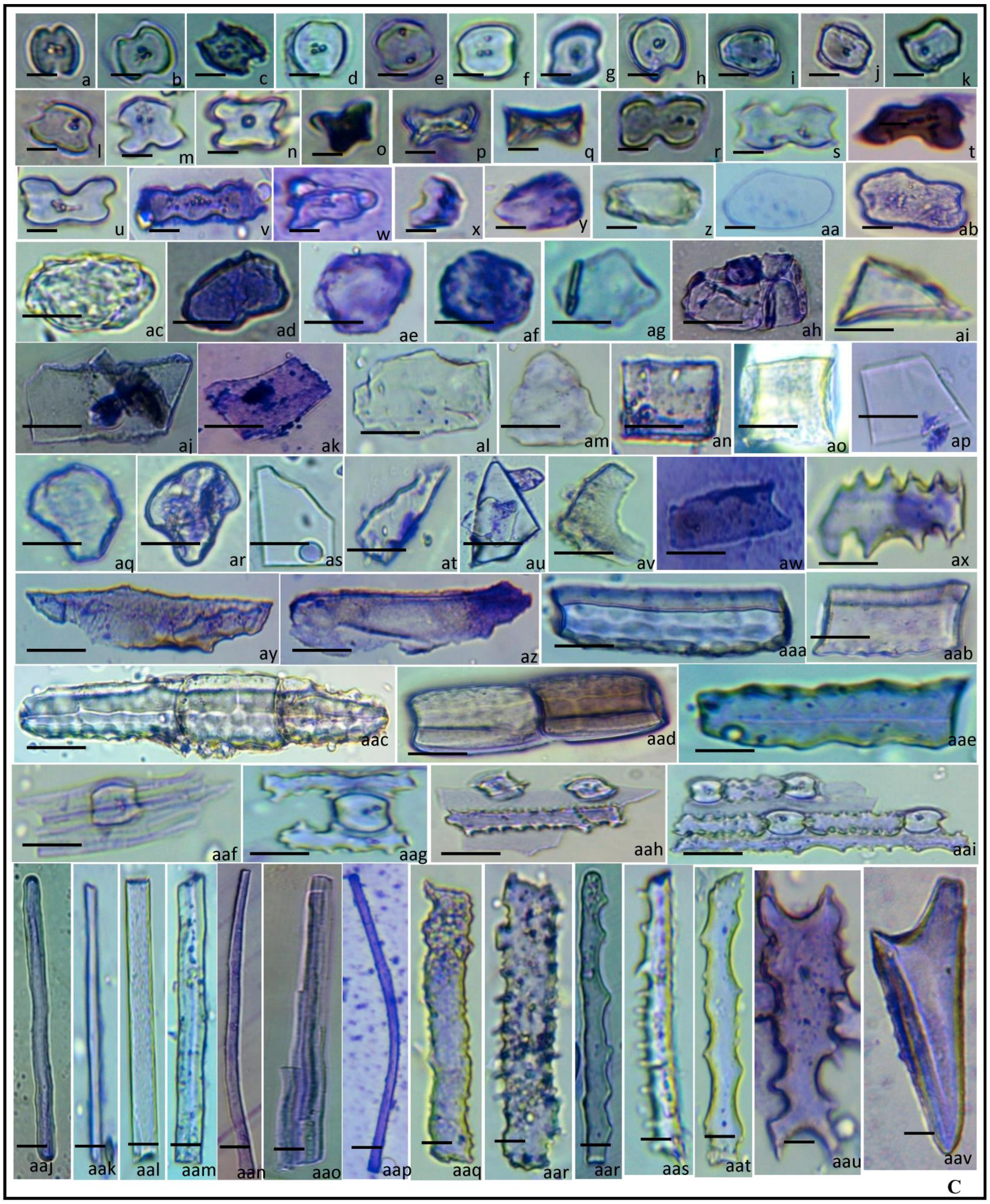
a
b
c
d
e
f
g
h
i
j
k
l
m
n
o
p
q
r
s
t
u
v
w
x
y
z
aa
ab
ac
ad
ae
af
ag
ah
ai
aj
ak
al
am
an
ao
ap
aq
ar
as
at
au
av
aw
ax
ay
az
aaa
aab
aac
aad
aae
aaf
aag
aah
aai
aaj
aak
aal
aam
aan
aao
aap
aaq
aar
aar
aas
aat
aau
aav
C

Fig. 3C

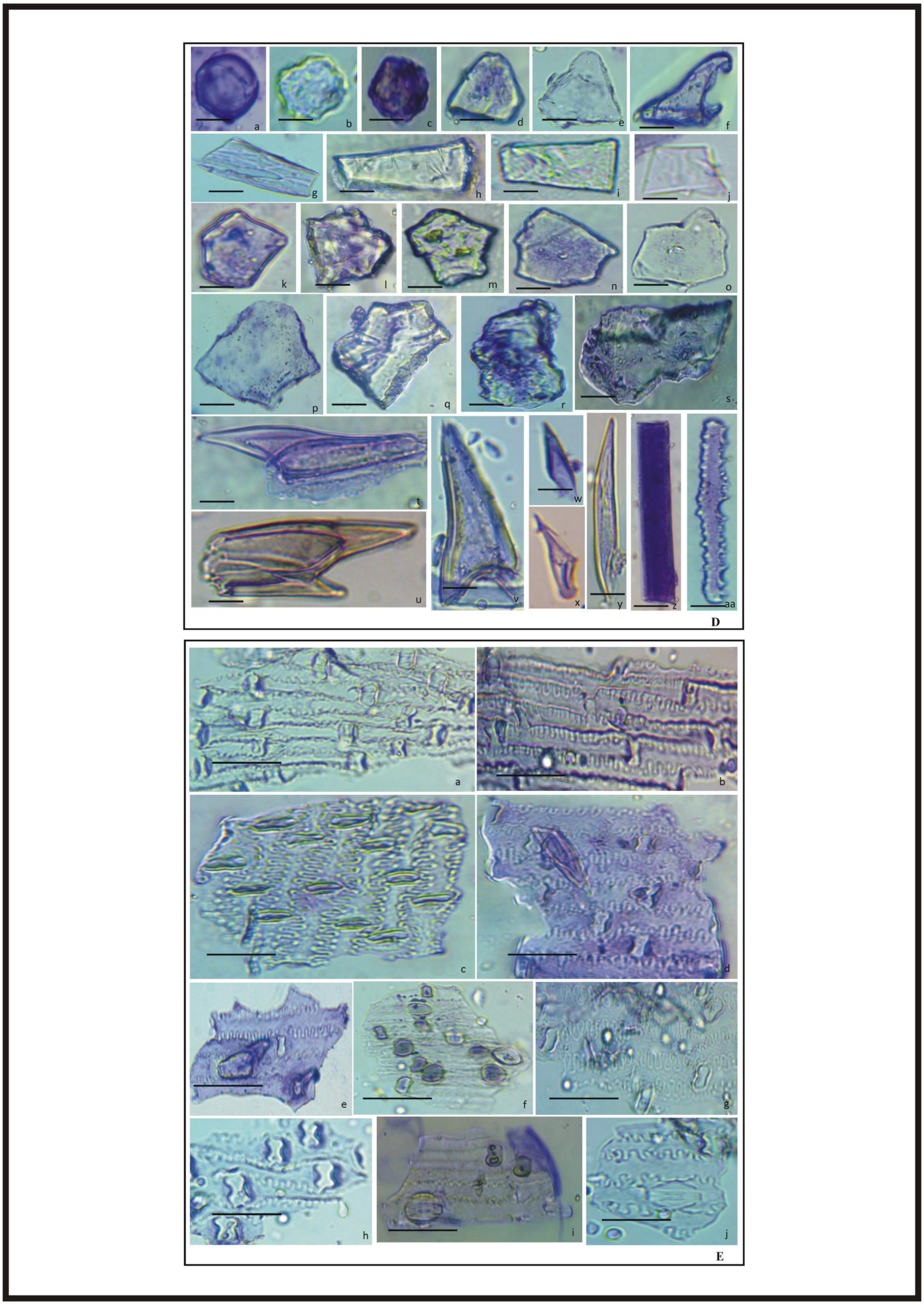

Fig. 3D and E

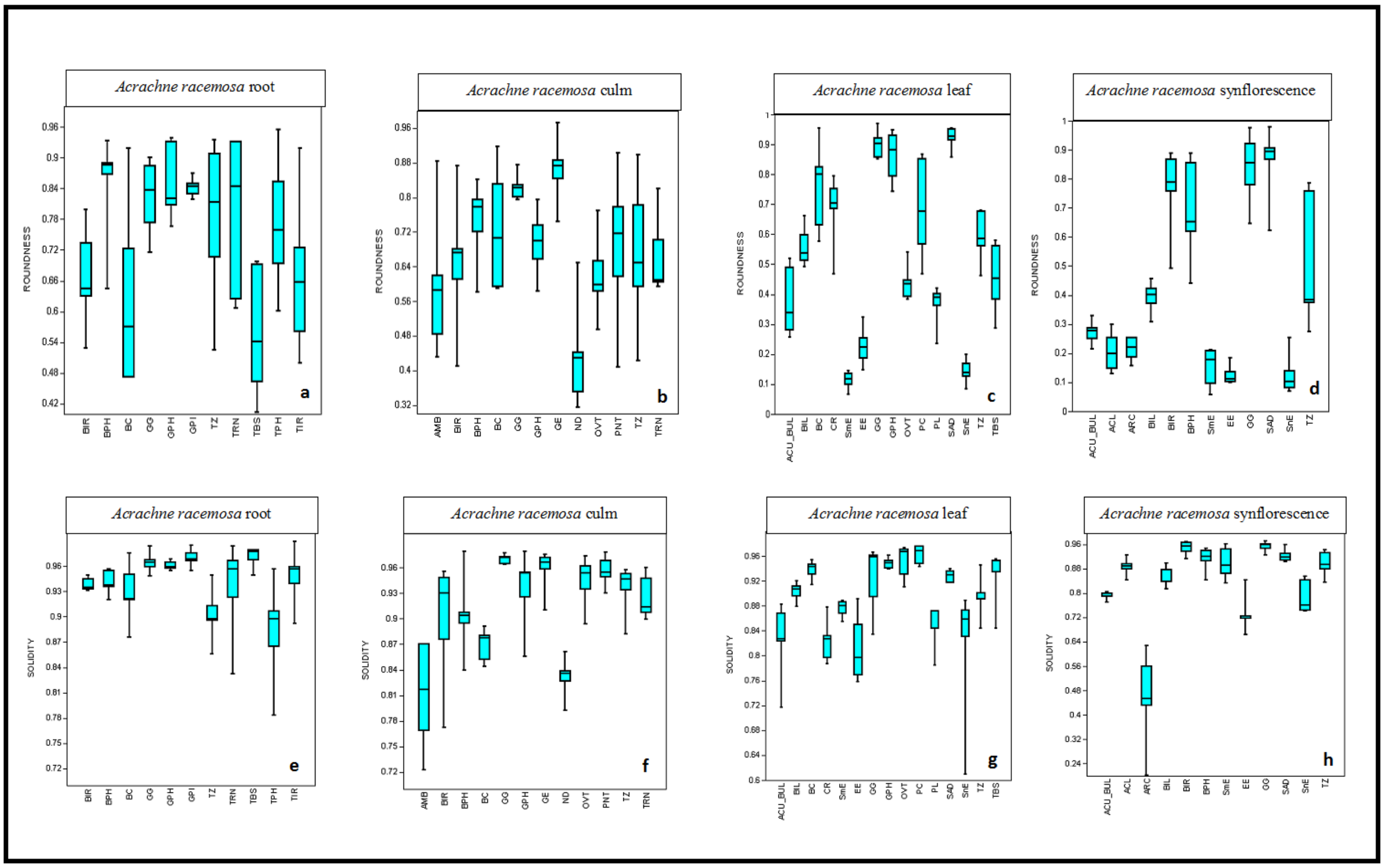

Acrachne racemosa root
ROUNDNESS
a
Acrachne racemosa culm
ROUNDNESS
b
Acrachne racemosa leaf
ROUNDNESS
c
Acrachne racemosa synflorescence
ROUNDNESS
d
Acrachne racemosa root
SOLIDITY
e
Acrachne racemosa culm
SOLIDITY
f
Acrachne racemosa leaf
SOLIDITY
g
Acrachne racemosa synflorescence
SOLIDITY
h

Fig. 4

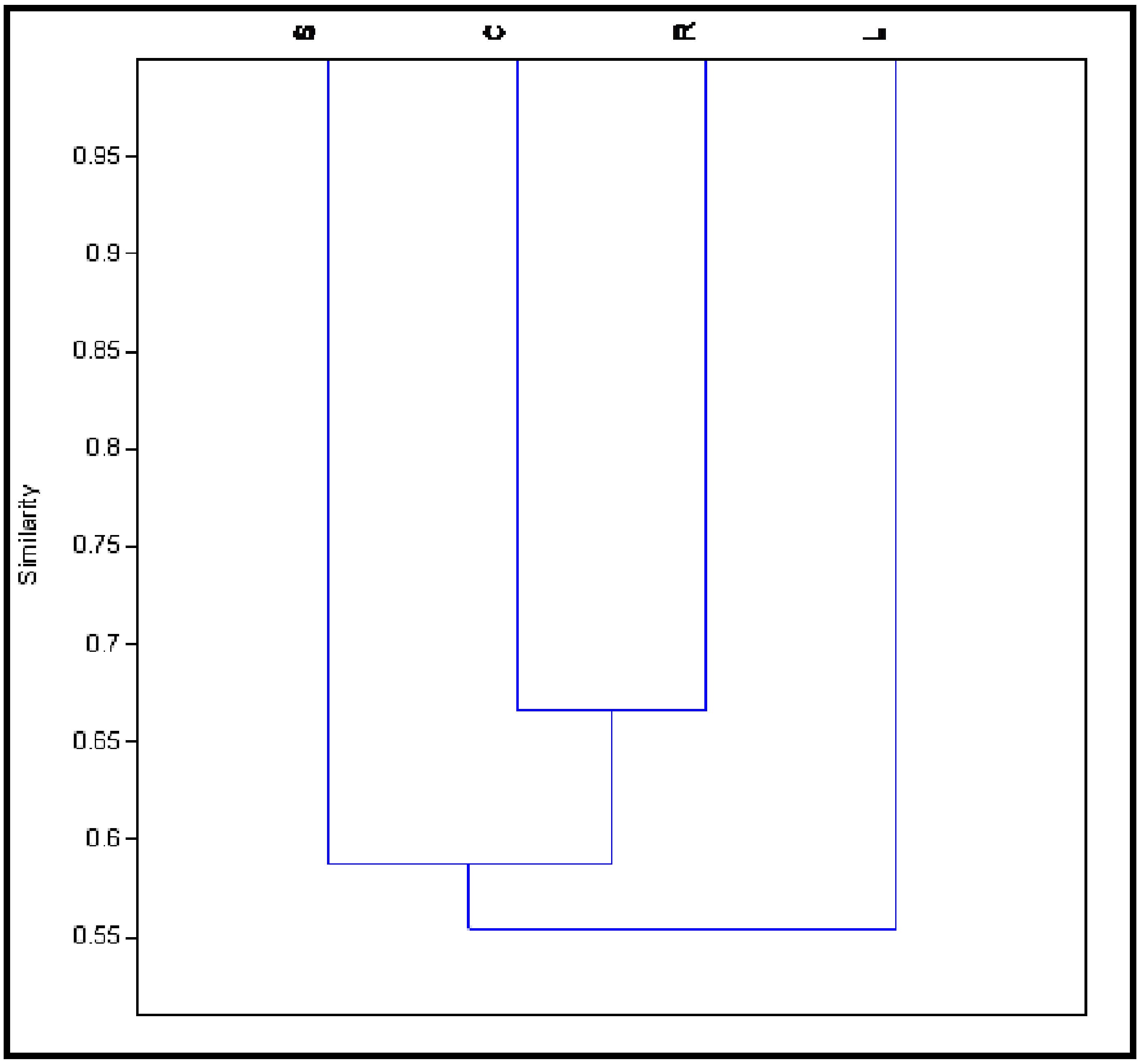

Fig. 5

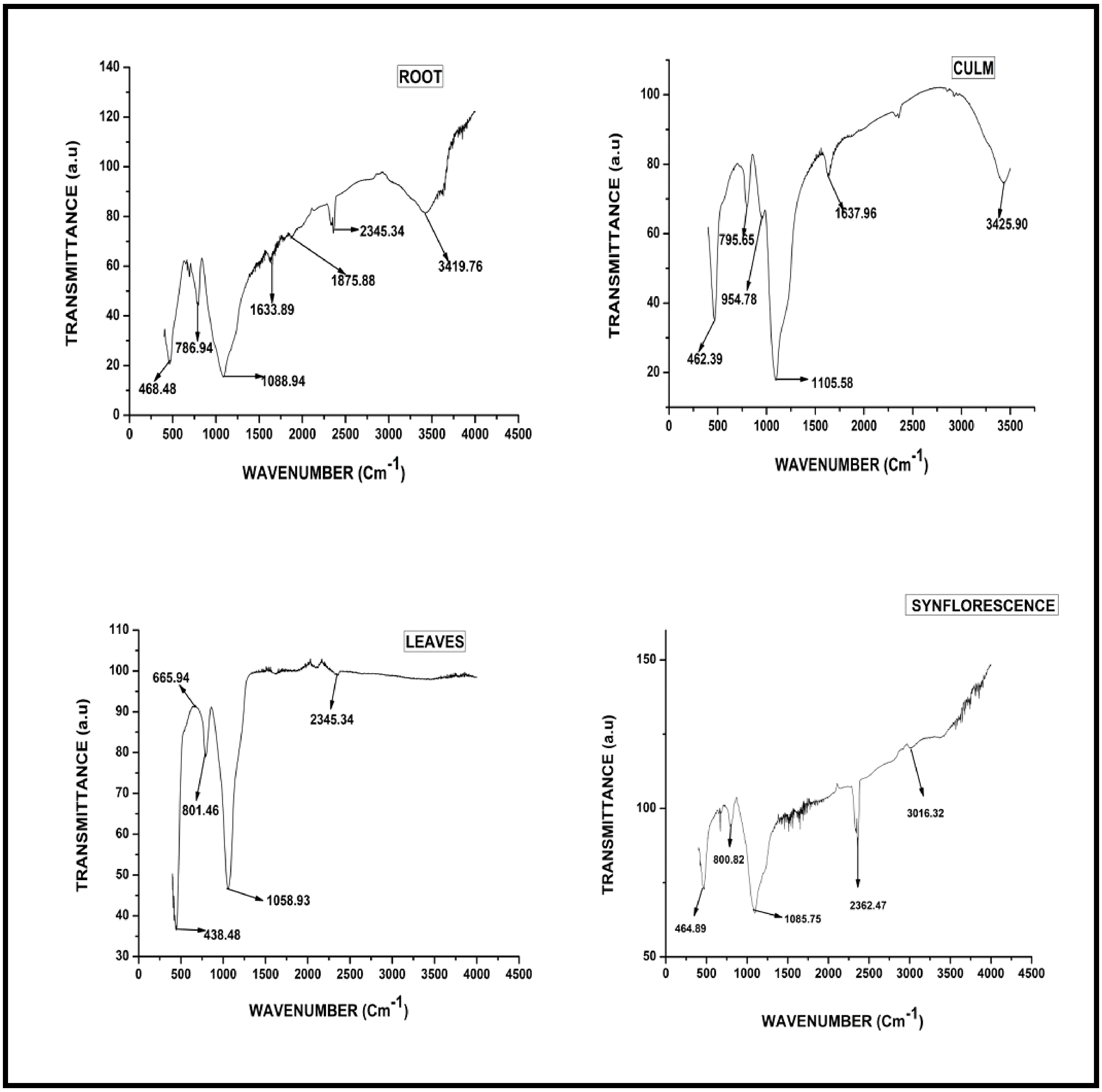
ROOT
TRANSMITTANCE (a.u)
WAVENUMBER (Cm-1)
468.48
786.94
1088.94
1633.89
1875.88
2345.34
3419.76
CULM
795.65
954.78
462.39
1105.58
1637.96
3425.90
LEAVES
665.94
801.46
1058.93
438.48
2345.34
SYNFLORESCENCE
464.89
800.82
1085.75
2362.47
3016.32

Fig. 6

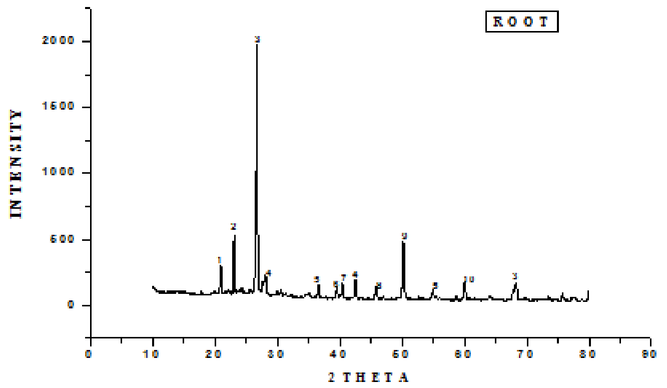

1. Hexagonal Quartz low primitive (1,0,0)
2. Orthorhombic tridymite end-centered (1,1,1)
3. Hexagonal Quartz primitive (0,1,1 ; 0,3,1)
4. Anorthic primitive (0,1,1 ; 1,2,2)
5. Hexagonal primitive (4,1,1 ; 0,2,2)
6. Monoclinic coesite end-centered (1,5.1)
7. Monoclinic end-centered (1,3,1)
8. Tetragonal stishovite primitive (1,1,1)
9. Orthorhombic body-centered (2,0,4)
10. Tetragonal cristobalite alpha primitive (1,1,4)

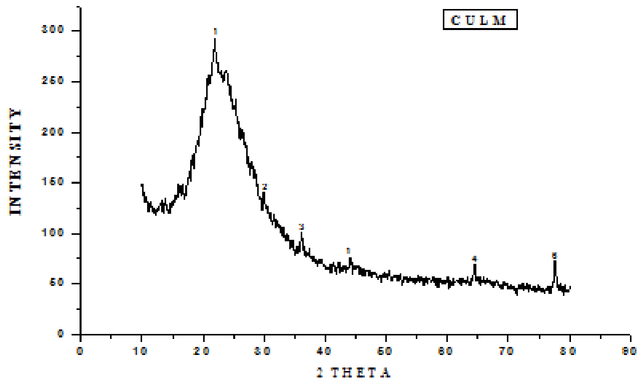

1. Tetragonal cristobalite alpha primitive (1,0,0 ; 1,0,3)
2. Anorthic primitive (1,1,1)
3. Monoclinic end-centered (2,2,0)
4. Tetragonal body-centered (6,0,0)
5. Hexagonal Quartz primitive (2,2,0)

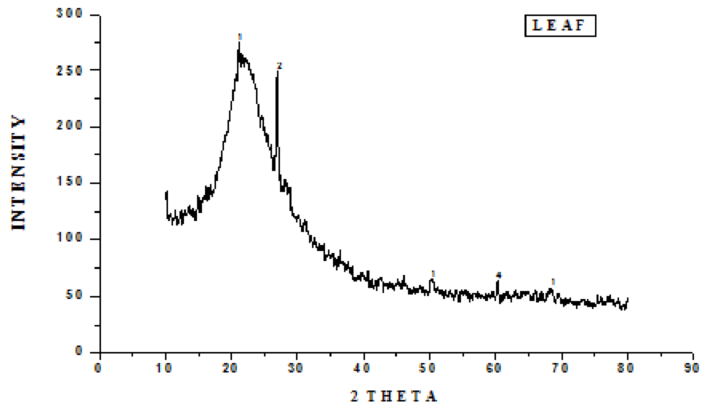

1. Hexagonal Quartz primitive (1,0,0 ;1,1,2 ; 0,3,1)
2. Anorthic primitive (1,1,0)
3.Tetragonal cristobalite alpha primitive (3,1,1)

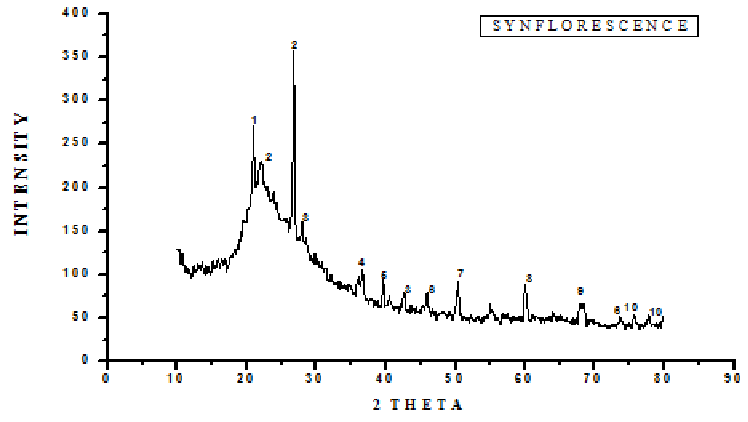

1. Hexagonal Quartz low primitive (1,0,0)
2. Orthorhombic primitive (1,1,0 ; 0,2,0)
3. Anorthic primitive (0,1,1 ; 1,2,2)
4. Hexagonal primitive (4,1,1)
5. Monoclinic coesite end-centered (1,5,1)
6. Tetragonal stishovite primitive (1,1,1 ; 2,2,1)
7. Monoclinic primitive (1,2,1)
8. Tetragonal cristobalite alpha primitive (1,1,4)
9. Hexagonal non-ambient pressure primitive (2,1,0)
10. Hexagonal Quartz primitive (3,0,2 ;2,2,0)

Fig. 7
