# Supplementary material for: Phytolith profile of Acrachne racemosa (B. Heyne ex Roem. & Schult.) Ohwi (Cynodonteae, Chloridoideae, Poaceae)
Source: PLoS One. 2022 Feb 11;17(2):e0263721. doi: 10.1371/journal.pone.0263721 (PMC8836352; doi:10.1371/journal.pone.0263721)
Supplement: Attachment — Submitted filename: Response to reviewers.docx [file pone.0263721.s006.docx]

Subject: Submission of Revised Manuscript number PONE-D-21-09972R2 entitled 'Phytolith profile of *Acrachne racemosa* (Heyne ex Roem. & Schult.) Ohwi (Cynodonteae, Chloridoideae, Poaceae)'.

Respected Sir/Madam,

It is submitted that the manuscript no. PONE-D-21-09972R1 'Phytolith profile of *Acrachne racemosa* (Heyne ex Roem. & Schult.) Ohwi (Cynodonteae, Chloridoideae, Poaceae)' has been revised thoroughly as per the suggestions and comments of the Editor and reviewers. The manuscript has been revised under the supervision of Dr. Amarjit Singh Soodan.

**Response to Reviewers**

Line 68: change “ha-1” to “ha^-1^”. [superscript (-1)]. Follow this style throughout the whole MS wherever it exists

Ans: “ha-1” has been corrected to “ha^-1^” (Line 69) and this style has been followed throughout the whole MS wherever it exists in the revised manuscript.

Line 122: change “50ml” to “50 mL”. change “80◦C” to degree symbol “80 °C”. Follow this style throughout the whole MS where it exists

Ans: “50ml” has been corrected to “50 mL” and 80◦C to 80 °C (Line 123) and this style has been followed throughout the whole MS wherever it exists in the revised manuscript.

Line 145: change letter “x” to symbol of cross “×”

Ans: Letter “x” has been changed to symbol of cross “×” in the revised manuscript (Line 146).

Line 221: change “bar =40” to “bar = 40”. Add spaces before and after the symbol “=”. Follow this style throughout the whole MS where it exists

Ans: “bar =40” has been changed to “bar = 40” (Line 222) and the spaces has been added before and after the symbol “=”. This style has been followed throughout the whole MS wherever it exists in the revised manuscript.

Table 2a, 2b, 2c, 2d: Add spaces before and after the symbol “±”.

Ans: Spaces have been added before and after the symbol “±” in table 2a, 2b, 2c, 2d in the revised manuscript.

Line 367: delete the space after the slash “/”

Ans: Space has been deleted in the revised manuscript (Line 363).

Line 443: add spaces before and after the symbol “>” and “˂”

Ans: Spaces have been added before and after the symbol “>” and “˂” in the revised manuscript (Line 410).

**References:** **review your reference list to ensure that it is complete and correct**

I have reviewed and complete the references as per the journal style in the revised manuscript and there is no retracted paper.
