# Supplementary material for: Phytolith profile of Acrachne racemosa (B. Heyne ex Roem. & Schult.) Ohwi (Cynodonteae, Chloridoideae, Poaceae)
Source: PLoS One. 2022 Feb 11;17(2):e0263721. doi: 10.1371/journal.pone.0263721 (PMC8836352; doi:10.1371/journal.pone.0263721)
Supplement: Attachment — Submitted filename: Reviewer comments.docx [file pone.0263721.s003.docx]

**Manuscript Number: PONE-D-21-09972**

**Full Title: Phytolith profile of Acrachne racemosa (Heyne ex Roem. & Schult.) Ohwi (Cynodonteae, Chloridoideae, Poaceae)**

**The manuscript presents a very good, original & exhaustive piece of work done by the authors and the work has multidisciplinary applications. The phytoliths are very important taxonomic tools vis-à-vis aid in the reconstruction of paleo-environments and prediction of climate changes.**

**However, the manuscript needs a major revisionary work to make it worth accepting in PLOSONE Journal. Following are queries/points which need to be addressed and necessary changes should be incorporated in the manuscript accordingly.**

1. **The authority of the taxon under investigation is incomplete.**
2. **The abstract of the manuscript is not coherent & robust and needs to be re-written.**
3. **The manuscript entitled “A worldwide phylogenetic classification of the Poaceae (Gramineae) II: An update and a comparison of two 2015 classifications.” which has been cited in the abstract section as [2] has been wrongly interpreted as the number of sub-tribes under the tribe cyanodontae are 21 not 18.**
4. **The introduction needs a systematic arrangement of sentences as it lacks logical sequence of sentences.**
5. **The manuscript should be supplemented by the herbarium sheet of the taxon under investigation.**
6. **The methodology should be made brief and concise as the manuscript is already very much voluminous and lengthy.**
7. **The methodology for FTIR is not clear.**
8. **The methodology for XRD is not mentioned instead use of XRD is presented.**
9. **Silica is an amorphous material but you have observed the crystalline phases in XRD studies. Explain the reasons for this shift from amorphous to crystalline behavior of silica.**
10. **There is repetition of sentences like----** *Acrachne racemosa* (Heyne. Ex roem. & Schult.) Ohwi is an annual grass having erect, simple or branched, tufted culms and…..**the repetitions of sentences should be avoided.**
11. **There is no need of the Table 1 an this information already exists in literature vis-à-vis on various taxonomic compilations and e-floras.**
12. **Explain the reasons for the accumulation of more silica content in leaves (The silica is absorbed by the plant roots from the soil and is then carried along the transpirational stream to different plant parts where it is subsequently deposited----correlate the silica content of various plant parts in relation to this statement)**
13. **The presence of saddle shaped phytoliths is characteristic feature of subfamily cyanodonteae……no such mention has been made in the manuscript despite of the presence of saddles in leaf peelings.**
14. **The results of In-situ locations of phytoliths should be rewritten so as to make then concise, brief and meaningful.**
15. **In Fig.2…Adaxial (B) should be presented before the Abaxial (A).**
16. **Incorporate the Fig. numbers in the morphometry tables of phytoliths against the each phytolith morphotype presented in morphometric tables.**
17. **The morphotypes in tables with and without data does not correlate with the data presented in the text. There is lot of ambiguity in the morphotype data (In Table 2A 27 morphotypes are mentioned from root.....data of only 11 morphotypes is presented and in text only 24 morphotypes in mentioned in total from the root).**
18. **) There is also a lot of ambiguity in the figure number of morphtypes between the text and the legend of the figures.**
19. **No methodology for the study of undulations of phytoliths is presented. Mention the respective parts of the synflorescence from which these undulation patterns were reported (glumes, lemma, palea etc)…if not worked out in the present study…….give support from literature**
20. **The cumulative frequency data of all the phytolith morphotypes from a plant part should be equal to 100 or 100% but the values are lower that 100% which is inappropriate.**
21. **Present the FTIR RESULTS in a tabulated form.**
22. **The transition of amorphous silica to crystalline form does not take place at 550 ^0^C but at a temperature higher than 1000^0^C. Support your finding from literature and look for some other reasons that may have brought about phase shift from amorphous to crystalline form of silica.**
23. **NO mention is made in the manuscript about the method of deciphering of XRD peaks. Have you made use of literature in peak deciphering or any software package and if so send us the working pics of the methods of peak deciphering for surety of the results presented in the manuscript.**

**Thanks and best of luck**
